# Distributional Replication

**DOI:** 10.3390/e23081063

**Published:** 2021-08-17

**Authors:** Brendan K. Beare

**Affiliations:** School of Economics, University of Sydney, Sydney, NSW 2006, Australia; brendan.beare@sydney.edu.au

**Keywords:** distributional replication, sieve estimation, hedge fund replication

## Abstract

A function which transforms a continuous random variable such that it has a specified distribution is called a replicating function. We suppose that functions may be assigned a price, and study an optimization problem in which the cheapest approximation to a replicating function is sought. Under suitable regularity conditions, including a bound on the entropy of the set of candidate approximations, we show that the optimal approximation comes close to achieving distributional replication, and close to achieving the minimum cost among replicating functions. We discuss the relevance of our results to the financial literature on hedge fund replication; in this case, the optimal approximation corresponds to the cheapest portfolio of market index options which delivers the hedge fund return distribution.

## 1. Introduction

Suppose that *X* and *Y* are random variables. In this paper we consider estimating a function θ such that θ(X) and *Y* have the same distribution. Such a function is said to be a *replicating function*. Typically, there are many different replicating functions for a given pair of random variables *X* and *Y*. We suppose that to each function θ there corresponds a “price”, denoted p(θ), and we seek to estimate the replicating function θ for which p(θ) is as small as possible. That is, we seek to estimate the cheapest replicating function for a given *X* and *Y*. To estimate this function from a sample of realizations of *X* and *Y*, we first obtain an estimate of the set of all replicating functions. The estimated set is formed by choosing a rich but manageable class of functions (i.e., a sieve space) and taking all those functions θ in that class for which the distance between the empirical distributions of θ(X) and *Y* is small. Our estimate of the cheapest replicating function is then obtained by minimizing *p* over the estimated set of replicating functions.

Our research is motivated by a literature in applied finance on “hedge fund replication”. The hedge fund replication literature is concerned with the possibility of achieving financial returns that resemble those of a particular hedge fund, fund of hedge funds, or index of hedge funds, by engaging in an investment strategy that does not involve a direct investment in the fund or funds in question. Ideally, the replicating strategy should involve trading assets that are highly liquid, thereby avoiding the barriers to entry, lock-in periods and high fees that are characteristic of hedge fund investments. Several major investment banks have launched hedge fund replication products, including Goldman Sachs and Merrill Lynch in 2006 and J.P. Morgan in 2007 [1]. Hedge fund replication strategies have also attracted the attention of the popular press, with articles appearing in *The Wall Street Journal* [2] and *The New Yorker* [3], among other outlets. Simonian and Wu [4] have recently described the proliferation of hedge fund replication strategies in investing as a “cottage industry”.

There are two broad streams of the hedge fund replication literature. In one stream, researchers have considered the direct approximation of hedge fund returns by investing in a portfolio of other assets. By direct approximation, we mean that the returns from the selected portfolio should be close to the hedge fund returns with high probability. Typically, the replicating strategy amounts to estimating a factor model for hedge fund returns, and then investing directly in the factors rather than in the hedge fund. Hasanhodzic and Lo [5] and Simonian and Wu [4] are representative of this stream of research. The second stream of the hedge fund replication literature is concerned with the distributional approximation of hedge fund returns, rather than their direct approximation. The aim here is to create a trading strategy that generates returns with the same statistical distribution as the hedge fund returns. This is a more modest goal than direct approximation, because in any given period the return generated by the replicating strategy need not resemble the return from the hedge fund. Key papers in this stream of the hedge fund replication literature include Amin and Kat [6], Kat and Palaro [7,8], and Kat [1]. The results in this paper concern the approach taken by these authors. Our aim is not to provide statistical methods ready to be applied to data, but rather to develop a mathematical framework for thinking about distributional replication.

Suppose that *X* represents the payoff after one month from a $1 investment in a market index, while *Y* represents the payoff after one month from a $1 investment in a hedge fund. Amin and Kat [6] propose to estimate a function θ such that θ(X) and *Y* have the same distribution function. Given a sample of *n* realizations of *X* and *Y*, their estimated replicating function is ${\hat{\theta }}_{n}={\hat{Q}}_{n}^{Y}\circ {\hat{F}}_{n}^{X}$, where ${\hat{Q}}_{n}^{Y}$ is an estimate of $Q^{Y}$, the quantile function of *Y*, and ${\hat{F}}_{n}^{X}$ is an estimate of $F^{X}$, the distribution function of *X*. Assuming continuity of $F^{X}$, the random variable $Q^{Y}\left(F^{X}\left(X\right)\right)$ has the same distribution as *Y*, implying that $Q^{Y}\circ F^{X}$ is a replicating function. We might therefore expect ${\hat{\theta }}_{n}\left(X\right)$ and *Y* to have similar distributions in large samples. The estimated function ${\hat{\theta }}_{n}$ can be thought of as describing the payoff after one month of a derivative security written on the market index. Under suitable conditions, this payoff can be achieved using a continuously rebalanced self-financed portfolio of market shares and cash, as in the hedging strategy used to justify the celebrated Black-Scholes-Merton option pricing formula [9,10]. We let p(θ) denote the start-up cost of a hedging strategy with payoff θ(X), and refer to this quantity as the price of θ.

It need not be the case that p(θ)=1 when θ is a replicating function. This is because the distributional equivalence of θ(X) and *Y* does not imply the existence of an arbitrage opportunity when their initial investment costs differ. Indeed, two replicating functions need not have the same price. Amin and Kat [6] aim to estimate the particular replicating function $Q^{Y}\circ F^{X}$ because it is an increasing function of the market payoff *X*. In Dybvig [11,12] and Beare [13], it is shown under very general conditions that, given a collection of payoff functions that all achieve the same payoff distribution, the cheapest such function must allocate payoffs to states as a nonincreasing function of the state prices. Amin and Kat [6] observe that in a Black-Scholes world, the state price density (with respect to the true probability measure over states) is inversely related to *X*. Thus, the cheapest replicating function must be a nondecreasing function of *X*.

A key difference between the approach to distributional replication proposed in this paper, and the approach taken by Amin and Kat [6], is that we do not assume that the cheapest replicating function is nondecreasing. Instead, we search for the cheapest replicating function over a large space of functions, many of which are not monotone. Empirically, there is good reason to believe that the cheapest replicating function will not be monotone. Jackwerth [14] and Brown and Jackwerth [15] argue that the state price density (in their terminology, pricing kernel) implied by S&P500 options with one month to expiry changed dramatically after the stock market crash of 1987, becoming nonmonotone with respect to the return on the S&P500 index. See, in particular, Figure 2 in [15], in which the state price density is an increasing function of the market return for monthly return levels between approximately −3% and 3%, and decreasing elsewhere. Other empirical studies of the relationship between the state price density and market returns have largely confirmed that it is often nonmonotone [16,17,18,19,20,21,22]. See also [23] for a discussion of the relevance of such nonmonotonicity for constructing density forecasts of market returns. If the relationship between the state price density and the market return is not monotone, then the results of Dybvig [11,12] and Beare [13] imply that the cheapest replicating function θ will not be monotone. In this case, the approach to distributional replication taken here is advantageous.

There is a second major conceptual difference between the approach to distributional replication taken here, and the approach taken by Amin and Kat [6]. Amin and Kat propose to implement the desired payoff function θ by engaging in a continuous time hedging strategy, trading market shares and cash. In this paper, we propose to approximate θ by investing in a portfolio of European put and call options written on the market index at various strike prices. The portfolio may also include the market index itself, and risk-free zero-coupon bonds. A key advantage of our approach is that the price of the payoff function θ corresponding to such a portfolio may be calculated directly from observed option and bond prices. By comparison, Amin and Kat price θ by taking the risk neutral expected payoff of θ(X) under Black-Scholes conditions, and they require Black-Scholes conditions to hold in order for their hedging strategy to achieve the desired payoff. The empirical limitations of the Black-Scholes pricing model have been extensively documented. We avoid these difficulties by confining ourselves to functions θ for which the market price is directly observable, and which may be implemented in practice by investing directly in a portfolio of actively traded securities.

We embed our approach in the statistical framework of sieve estimation by assuming that the set of strike prices at which options may be traded becomes more dense as the sample size *n* increases, at a controlled rate. The payoff functions achievable using portfolios of this kind are continuous piecewise linear functions, with kinks at the allowable strike prices. We control the entropy (complexity) of this class of functions using the notion of VC-dimension [24], and are thereby able to bring the machinery of empirical process theory to bear in analyzing the asymptotic properties of our technique. The use of option payoff functions to form the basis for a sieve space is not entirely without precedent. Option payoffs appear as activation functions in the regularized neural network model studied by Corradi and White [25]: take m=2 in their Equation (4.1). Those authors do not, however, explicitly discuss the connection to option payoffs and portfolio choice.

The approach taken by Amin and Kat [6], and in this paper, aims to replicate the univariate distribution of *Y*. Typically, the joint distribution of θ(X) with any other asset payoff will differ from the joint distribution of *Y* and that asset payoff. In particular, the joint distribution of θ(X) and the market payoff *X* will differ from the joint distribution of *Y* and *X*, and for this reason we cannot expect investors to find θ(X) to be a perfect substitute for *Y* in general. Intuitively, if the correlation between *X* and *Y* is lower than the correlation between *X* and θ(X), risk-averse investors may prefer a balanced portfolio formed from *X* and *Y* to a similar portfolio formed from *X* and θ(X). In response to this issue, Kat and Palaro [7,8] extend the approach of Amin and Kat [6] to the replication of bivariate distributions. They introduce a “reserve asset” with payoff *Z*, and seek to find a bivariate function θ such that the joint distribution of θ(X,Z) and *X* is the same as the joint distribution of *Y* and *X*. This replicating payoff function is implemented in practice using a continuously rebalanced portfolio formed by trading market shares, cash, and the reserve asset. We do not follow that approach in this paper, in part because it is generally not feasible to approximate a wide class of bivariate functions using a portfolio formed from options written on individual assets. Confining ourselves to the replication of univariate distributions may not seem unreasonable if we modify our interpretation of the random variable *Y*. Rather than representing the payoff from a $1 investment in a hedge fund, *Y* could represent the payoff from a $1 investment in a portfolio partly invested in the hedge fund and partly in the market index. Amin and Kat [6] take this approach in their empirical study of hedge fund efficiency. More generally, *Y* could be the payoff from a $1 investment in a portfolio formed from any number of arbitrary assets. If θ(X) has the same distribution as *Y*, and the price of θ is less than $1, an investor should prefer to invest in the replicating portfolio.

The remainder of this paper is structured as follows. In Section 2 and Section 3 we develop a general approach to the estimation of replicating functions, without explicit reference to the financial application that serves as our motivation. In Section 2 we provide some basic mathematical tools for dealing with the notion of replicating functions, while in Section 3 we discuss the statistical estimation of replicating functions using the method of sieves. In Section 4 we explain how the mathematical material in Section 2 and Section 3 can be applied to the problem of hedge fund replication. Section 5 outlines some areas for future research, and concludes. Throughout the paper, there are several numbered assumptions and propositions. In the statement of each proposition, it should be understood that all assumptions introduced prior to the proposition hold. Proofs of all numbered propositions may be found in Appendix A.

## 2. Replicating Functions

In this section we formally introduce the notion of a replicating function. We construct a pseudometric on the set of Borel measurable functions mapping the support of one random variable to the support of another, and we define a criterion function that identifies the set of replicating functions. Some useful results relating to these objects are given.

Let *X* and *Y* be real valued random variables, and let $P^{X}:B\left(R\right)\to \left[0,1\right]$ and $P^{Y}:B\left(R\right)\to \left[0,1\right]$ denote the probability measures corresponding to *X* and *Y*, where B(R) denotes the usual Borel σ-field on R. Let $F^{X}:R\to \left[0,1\right]$ and $F^{Y}:R\to \left[0,1\right]$ denote the distribution functions of *X* and *Y*. Let $R^{X}=\mathrm{cl}\left(\left\{x\in R:0<F^{X}\left(x\right)<1\right\}\right)$, and let $R^{Y}=\mathrm{cl}\left(\left\{y\in R:0<F^{Y}\left(y\right)<1\right\}\right)$; here, cl(A) denotes the Euclidean closure of a set A⊆R. The sets $R^{X}$ and $R^{Y}$ are intervals of the form [a,b], [a,∞) or (∞,b], with a,b∈R. We place the following condition on $F^{X}$ and $F^{Y}$.

**Assumption** **1.** *$F^{X}$ and $F^{Y}$ are continuous and strictly increasing on $R^{X}$ and $R^{Y}$ respectively.*

Assumption 1 is stronger than is required to establish all of the results in this paper, but it will be convenient for us to maintain Assumption 1 throughout. Under Assumption 1, the restriction of $F^{X}$ to $R^{X}$ is a continuous and strictly increasing function, and therefore uniquely defines a continuous and strictly increasing inverse function $Q^{X}:F^{X}\left(R^{X}\right)\to R^{X}$. We refer to this function as the quantile function of *X*. The quantile function of *Y*, denoted $Q^{Y}:F^{Y}\left(R^{Y}\right)\to R^{Y}$, is defined in the same way. Note that $F^{X}\left(R^{X}\right)$ and $F^{Y}\left(R^{Y}\right)$ are equal to (0,1], [0,1), [0,1] or (0,1), depending on whether *X* and *Y* are almost surely bounded above, below, both, or neither.

Let Θ denote the set of all Borel measurable functions $\theta :R^{X}\to R^{Y}$. Though Θ depends on *X* and *Y*, we do not make this dependence explicit in our notation. We are interested in those functions θ∈Θ for which θ(X) and *Y* have the same distribution.

**Definition** **1.** *A function θ∈Θ is called a replicating function for X and Y, or simply a replicating function or replicator, if $P^{X}\theta^{-1}B=P^{Y}B$ for all B∈B(R).*

Note that a replicating function does not describe a relationship between *X* and *Y* in the usual sense. θ(X) and *Y* may be perfectly correlated, or independent. All that matters is that they have the same marginal distribution. We will let $\Theta^{*}$ denote the set of all replicating functions for *X* and *Y*. Again, the dependence of $\Theta^{*}$ on *X* and *Y* is not made explicit in our notation.

Our first result concerns the cardinality of $\Theta^{*}$.

**Proposition** **1.** $\Theta^{*}$ is uncountably infinite. Moreoever, there exists an uncountable subset of $\Theta^{*}$ in which no two functions are equal on a set of positive $P^{X}$-measure.

**Remark** **1.** One example of a replicating function is the composition $Q^{Y}\circ F^{X}$, restricted to $R^{X}$. Clearly, if $F^{X}$ is not continuous and $F^{Y}$ is continuous, so that Assumption 1 is violated, $\Theta^{*}$ is empty.

**Figure 1:**
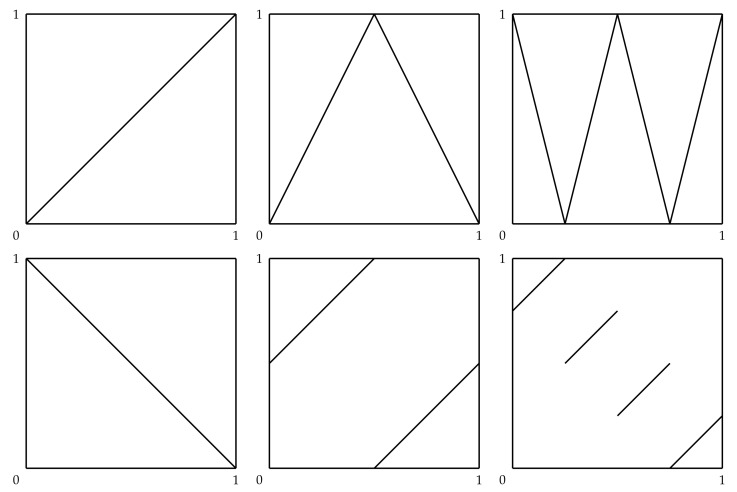
Examples of replicating functions when X,Y∼U(0,1).

We will sometimes find it helpful to consider the special case where *X* and *Y* are both distributed uniformly on the unit interval. In this case, the composition $\theta =Q^{Y}\circ F^{X}$ restricted to $R^{X}=\left[0,1\right]$ is the identity function, θ(x)=x. Another simple example of a replicating function is θ(x)=1−x, restricted to [0,1]. Graphs of these functions, and of four other replicating functions, are provided in Figure 1. We will let $\tilde{\Theta }$ denote the set of all Borel measurable functions θ:[0,1]→[0,1], and let ${\tilde{\Theta }}^{*}$ denote the set of functions in $\tilde{\Theta }$ that are replicators when X,Y∼U(0,1).

As an aid to visualizing the functions in ${\tilde{\Theta }}^{*}$, a reader familiar with the concept of local time may find it helpful to think of each function $\theta \in \tilde{\Theta }$ as a (nonrandom) stochastic process on the unit interval. The functions $\theta \in {\tilde{\Theta }}^{*}$ are precisely those for which the local time at *y* is equal to one for each y∈(0,1). That is, for $\theta \in \tilde{\Theta }$, we have $\theta \in {\tilde{\Theta }}^{*}$ if and only if

$$\lim_{\varepsilon ↓0}\frac{1}{2\varepsilon }\int_{0}^{1}1\left(|\theta \left(x\right)-y|\leq \varepsilon \right)dx=1$$

for each y∈(0,1). This can be shown by observing that the above limit is equal to the derivative of the distribution function of θ(X) at *y* when X∼U(0,1).

We now introduce a pseudometric *d* on Θ. For $\theta_{0},\theta_{1}\in \Theta$, let d:Θ×Θ→R be given by

$$d\left(\theta_{0},\theta_{1}\right)=\int_{R^{X}}\left|F^{Y}\left(\theta_{1}\left(x\right)\right)-F^{Y}\left(\theta_{0}\left(x\right)\right)\right|dF^{X}\left(x\right).$$

It is obvious that *d* satisfies the four axioms for a pseudometric: nonegativity, symmetry, the triangle inequality, and the requirement that d(θ,θ)=0 for all θ∈Θ. *d* is not a metric because we will have $d(\theta_{0},\theta_{1})=0$ when $\theta_{0}$ and $\theta_{1}$ are equal on a set of $P^{X}$-measure one, even if the two functions are distinct. Note that when X,Y∼U(0,1), *d* corresponds to the usual $L_{1}$-seminorm for functions on [0,1]. When *X* and *Y* are not uniform, $d(\theta_{0},\theta_{1})$ is equal to the $L_{1}$ distance between the deformed functions $F^{Y}\circ \theta_{0}\circ Q^{X}$ and $F^{Y}\circ \theta_{1}\circ Q^{X}$.

We now introduce a nonnegative function M:Θ→R that is intended to quantify the extent to which a function θ∈Θ achieves distributional replication. For θ∈Θ, let $F^{X}\left(\cdot ;\theta \right)$ denote the distribution function of θ(X); that is, for y∈R and θ∈Θ, let $F^{X}\left(y;\theta \right)=P^{X}\theta^{-1}\left(-\infty ,y\right]$. Define

$$M\left(\theta \right)=\int_{R^{Y}}\left|F^{X}\left(y;\theta \right)-F^{Y}\left(y\right)\right|dF^{Y}\left(y\right).$$

Our pseudometric *d* endows *M* with a convenient smoothness condition. Specifically, *M* is Lipschitz continuous with respect to *d*, with Lipschitz constant no greater than one.

**Proposition** **2.** *For all $\theta_{0},\theta_{1}\in \Theta$, we have $|M\left(\theta_{1}\right)-M\left(\theta_{0}\right)|\leq d\left(\theta_{0},\theta_{1}\right)$.*

Our next result concerns the identification of the set of replicators $\Theta^{*}$ using the criterion function *M*. It states that the set of replicators $\Theta^{*}$ is precisely those functions θ∈Θ for which M(θ)=0.

**Proposition** **3.** $\Theta^{*}=\left\{\theta \in \Theta :M\left(\theta \right)=0\right\}$.

Propositions 2 and 3 jointly imply that, if $\theta_{1},\theta_{2},\ldots$ is a sequence of elements of Θ converging to some $\theta^{*}\in \Theta^{*}$ in the pseudometric *d*, then $M(\theta_{n})\to 0$ as n→∞. We would like to interpret this to mean that $\theta_{n}$ gets arbitrarily close to achieving distributional replication as *n* becomes larger. The next result makes this notion precise.

**Proposition** **4.** *Let $\theta_{1},\theta_{2},\ldots$ be a sequence of elements of* Θ. *Then, as n→∞, $M(\theta_{n})\to 0$ if and only if $F^{X}\left(y;\theta_{n}\right)\to F^{Y}\left(y\right)$ for each y∈R.*

**Remark** **2.** *Note that, since $F^{Y}$ is continuous, pointwise convergence of $F^{X}\left(\cdot ;\theta_{n}\right)$ to $F^{Y}\left(\cdot \right)$ is equivalent to the statement $P^{X}\theta_{n}^{-1}\Rightarrow P^{Y}$, where* “⇒” *denotes weak convergence of probability measures (see e.g., [26]), and $P^{X}\theta_{n}^{-1}$ is the measure on B(R) given by $P^{X}\theta_{n}^{-1}B=P^{X}\left\{x\in R^{X}:\theta_{n}\left(x\right)\in B\right\}$ for each B∈B(R). We could also write this statement as $\theta_{n}\left(X\right)\to_{d}Y$, where “$\to_{d}$” denotes convergence in distribution in the usual sense.*

Our final result of this section is a modification of Proposition 4 that allows $\theta_{1},\theta_{2},\ldots$ to be random elements. An obvious first step towards defining such random elements would be to introduce a σ-field on Θ; however, such an approach leads to complications relating to the measurability of θ(X) when θ and *X* are both random. We will need to require each of the random elements $\theta_{n}$, n∈N, to be a random element of some subspace $\Theta_{n}\subset \Theta$. Each subspace $\Theta_{n}$ will be equipped with a σ-field $T_{n}$ that is well behaved in the following sense.

**Definition** **2.** *Given a collection of functions $\Theta^{\prime }\subseteq \Theta$, an admissible structure for $\Theta^{\prime }$ is a σ-field $T^{\prime }$ of subsets of $\Theta^{\prime }$ such that the evaluation mapping (θ,x)↦θ(x) is a measurable map from $\left(\Theta^{\prime }\times R^{X},T^{\prime }⊗B\left(R^{X}\right)\right)$ to (R,B(R)).*

Definition 2 is a version of a definition of admissibility given in Section 5.2 of [27]. $B(R^{X})$ denotes the Borel σ-field on $R^{X}$, while the notation $T^{\prime }⊗B\left(R^{X}\right)$ refers to the product σ-field on $\Theta^{\prime }\times R^{X}$; that is, the σ-field on $\Theta^{\prime }\times R^{X}$ generated by sets of the form A×B, with $A\in T^{\prime }$ and $B\in B(R^{X})$. With Definition 2 in hand, we are now in a position to state the final result of this section.

**Proposition** **5.** *Let $\Theta_{1},\Theta_{2},\ldots$ be a sequence of subsets of* Θ, *and for each n∈N let $T_{n}$ be an admissible structure for $\Theta_{n}$ and let $P^{\theta_{n}}$ be a probability measure on $T_{n}$. Let $P^{\theta_{n}\left(X\right)}$ be the probability measure on B(R) given by $P^{\theta_{n}\left(X\right)}B=P^{\theta_{n}}⊗P^{X}\left\{\left(\theta ,x\right)\in \Theta_{n}\times R^{X}:\theta \left(x\right)\in B\right\}$ for each B∈B(R), where $P^{\theta_{n}}⊗P^{X}$ is the product measure on $T_{n}⊗B\left(R^{X}\right)$. Then, as n→∞, if $\int_{\Theta_{n}}MdP^{\theta_{n}}\to 0$ then also $P^{\theta_{n}\left(X\right)}\Rightarrow P^{Y}$.*

**Remark** **3.** *It is possible to rephrase Proposition 5 in a somewhat less precise fashion that may be easier to interpret. For each n∈N, we can think of the measure $P^{\theta_{n}}$ as corresponding to a random function $\theta_{n}$ taking values in $\Theta_{n}$. The measure $P^{\theta_{n}\left(X\right)}$ describes the distribution of $\theta_{n}\left(X\right)$ when $\theta_{n}$ and X are both random, and $\theta_{n}$ is independent of X. The statement $\int_{\Theta_{n}}MdP^{\theta_{n}}\to 0$ can be written as $EM(\theta_{n})\to 0$. Thus, the final statement of Proposition 5 could be written as follows: as n→∞, if $EM(\theta_{n})\to 0$ then also $\theta_{n}\left(X\right)\to_{d}Y$.*

## 3. Sieve Estimation of Replicating Functions

In this section we turn our attention to the statistical estimation of a replicating function using a sample of observations $\left\{\left(X_{i},Y_{i}\right):1\leq i\leq n\right\}$.

**Assumption** **2.** *$\left\{X_{i}:i\in N\right\}$ and $\left\{Y_{i}:i\in N\right\}$ are iid collections of real valued random variables defined on a complete probability space (Ω,F,P). Each $X_{i}$ has distribution function $F^{X}$, and each $Y_{i}$ has distribution function $F^{Y}$.*

**Remark** **4.** *The iid condition in Assumption 2 refers to the independence of $X_{i}$ and $X_{j}$, and of $Y_{i}$ and $Y_{j}$, when i≠j. $X_{i}$ and $Y_{j}$ may be dependent for any i,j.*

**Remark** **5.** *The assumption that (Ω,F,P) is complete will be useful later when we employ a result due to Stinchcombe and White [28] that provides conditions under which certain real valued functions on* Ω *are analytic (in the measure-theoretic sense). The interested reader may refer to that paper for the definition of an analytic function. When (Ω,F,P) is complete, real valued functions on* Ω *are analytic if and only if they are measurable.*

We wish to use our observed sample $\left\{\left(X_{i},Y_{i}\right):1\leq i\leq n\right\}$ to construct an estimate of a replicating function that has good properties when *n* is large. As was made clear in Proposition 1, the set of replicating functions is uncountably infinite in a nontrivial sense. We are thus confronted with the problem of partial identification: the distributional replication property does not uniquely identify the function we are seeking to estimate. The first step in our estimation procedure is to empirically discriminate between those functions that come close to achieving distributional replication, and those that do not. In the previous section, the function M:Θ→R was used to quantify the extent to which a function θ∈Θ achieves distributional replication. We will construct an empirical analogue to *M*. Given a sample of size *n*, let $F_{n}^{Y}:R\to \left[0,1\right]$ denote the empirical distribution function of *Y*, and for θ∈Θ let $F_{n}^{X}\left(\cdot ;\theta \right):R\to \left[0,1\right]$ denote the empirical distribution function of θ(X). That is,

$$\begin{array}{ccc} F_{n}^{Y}\left(y\right) & = & \frac{1}{n}\sum_{i=1}^{n}1\left(Y_{i}\leq y\right),\\ F_{n}^{X}\left(y;\theta \right) & = & \frac{1}{n}\sum_{i=1}^{n}1\left(\theta \left(X_{i}\right)\leq y\right). \end{array}$$

Let the function $M_{n}:\Theta \to R$ be defined by

$$M_{n}\left(\theta \right)=\int |F_{n}^{X}\left(y;\theta \right)-F_{n}^{Y}\left(y\right)|dF_{n}^{Y}\left(y\right)=\frac{1}{n}\sum_{i=1}^{n}\left|F_{n}^{X}\left(Y_{i};\theta \right)-F_{n}^{Y}\left(Y_{i}\right)\right|.$$

$M_{n}$ will serve as our empirical analogue to *M*. Note that we have suppressed the dependence of $F_{n}^{Y}$, $F_{n}^{X}$ and $M_{n}$ on ω∈Ω in our notation.

We would like $M_{n}$ to serve as a good approximation to *M* when *n* is large. Unfortunately, the space Θ is too rich for us to expect $M_{n}$ to be close to *M* uniformly over Θ. We shall instead consider the approximation of *M* by $M_{n}$ over a more manageable subset of the functions in Θ. We will consider a sequence of such subsets $\Theta_{1}\subseteq \Theta_{2}\subseteq \cdots$, with $\Theta_{n}$ becoming more complex as *n* grows, but at a slow enough rate to allow the uniform approximation error $\sup_{\theta \in \Theta_{n}}\left|M_{n}\left(\theta \right)-M\left(\theta \right)\right|$ to decay to zero in a suitable sense. Our approach may be regarded as a version of the method of sieve estimation. See [29] for a general discussion of sieve estimation in econometrics.

To control the entropy (complexity) of $\Theta_{n}$, we shall employ the notion of VC-major dimension. VC-major dimension is a characterization of complexity for classes of functions that is related to the notion of VC-dimension for classes of sets.

**Definition** **3.** *Let C be a collection of subsets of R. C is said to shatter a set of points $D=\{x_{1},\ldots ,x_{d}\}\subset R$, d∈N, if all $2^{d}$ subsets of D can be written as the intersection of D with some set in C. C is said to be a VC-class if, for some d∈N, C cannot shatter any set of size d. If C is a VC-class then the VC-dimension of C, written V(C), is defined to be the smallest d∈N for which no set of size d is shattered by C. If C is not a VC-class, we set V(C)=∞.*

Definition 3 is standard in the literature on empirical processes; see e.g., Section 2.6.1 in [30]. Building on Definition 3, we define the VC-major dimension of a subset of Θ as follows.

**Definition** **4.** *Consider a collection of functions $\Theta^{\prime }\subseteq \Theta$. A subset of R is said to be majorized by $\Theta^{\prime }$ if it can be written as $\left\{x\in R^{X}:\theta \left(x\right)>c\right\}$ for some $\theta \in \Theta^{\prime }$ and some c∈R. Let C denote the collection of all sets majorized by $\Theta^{\prime }$. We say that $\Theta^{\prime }$ is a VC-major class if C is a VC-class. The VC-major dimension of $\Theta^{\prime }$, written $V(\Theta^{\prime })$, is defined to be the VC-dimension of C.*

**Remark** **6.** *The definition of VC-major dimension should not be confused with that of VC-subgraph dimension, which also appears frequently in the empirical process literature; in general, the two are different. When $\Theta^{\prime }$ is the set of indicator functions of a collection of sets C, the VC-major dimension and VC-subgraph dimension of $\Theta^{\prime }$ are both equal to the VC-dimension of C. Sections 2.6.2 and 2.6.4 in [30] provide discussions of VC-subgraph and VC-major classes respectively.*

We will control the entropy of the spaces $\Theta_{n}$ by bounding the growth rate of their VC-major dimension. In addition, we will need to introduce some additional technical conditions to ensure the measurability of certain real valued functions on Ω. For $\Theta^{\prime }\subseteq \Theta$, let $B(\Theta^{\prime })$ denote the Borel σ-field on $\Theta^{\prime }$ induced by the pseudometric *d*.

**Assumption** **3.** *For each n∈N, $\Theta_{n}\subseteq \Theta$ is a nonempty VC-major class. Further, $B(\Theta_{n})$ is an admissible structure on $\Theta_{n}$, and $\left(\Theta_{n},B\left(\Theta_{n}\right)\right)$ is a Souslin measurable space.*

**Remark** **7.** *Refer to Stinchcombe and White [28] for the definition of a Souslin measurable space, and further discussion. Here, we note only that for $\left(\Theta_{n},B\left(\Theta_{n}\right)\right)$ to be a Souslin measurable space, it suffices that $\left(\Theta_{n},d\right)$ is a Polish metric space; that is, $\left(\Theta_{n},d\right)$ is a metric space that is topologically isomorphic to a complete separable metric space.*

The following result shows how the uniform approximation error $\sup_{\theta \in \Theta_{n}}\left|M_{n}\left(\theta \right)-M\left(\theta \right)\right|$ relates to $V(\Theta_{n})$.

**Proposition** **6.** *As n→∞, we have $E\sup_{\theta \in \Theta_{n}}\left|M_{n}\left(\theta \right)-M\left(\theta \right)\right|=O\left(\sqrt{V(\Theta_{n})/n}\right)$.*

**Remark** **8.** *In the proof of Proposition 6 it is established that $\sup_{\theta \in \Theta_{n}}\left|M_{n}\left(\theta \right)-M\left(\theta \right)\right|$ is a measurable function from (Ω,F) to (R,B(R)). Thus, our statement of Proposition 6 uses the ordinary expectation operator. It is common in the empirical process literature to see results of this kind expressed in terms of outer expectation; see e.g., Section 1.2 in [30].*

Proposition 6 indicates that, if $V\left(\Theta_{n}\right)=o\left(n\right)$, then when *n* is large we can use the empirical criterion function $M_{n}$ to distinguish between those functions in $\Theta_{n}$ that are close to achieving distributional replication, and those that are not. We have yet to address the issue of partial identification: there may be many functions in $\Theta_{n}$ that are close to achieving distributional replication. We wish to entertain the possibility that not all replicating functions are created equal. Let p:Θ→R be a function describing the “price” of each function θ∈Θ. Rather than seeking to estimate an arbitrary replicating function, we will seek to estimate a replicating function θ for which p(θ) is as small as possible.

**Assumption** **4.** *The function p:Θ→R is nonnegative, and continuous with respect to d.*

Loosely speaking, we seek to estimate the cheapest, or optimal, replicating function. The following result concerns the selection of our estimated function ${\hat{\theta }}_{n}$. In it, we make the random nature of $M_{n}$ explicit by writing $M_{n}$ as a function of both ω∈Ω and θ∈Θ.

**Proposition** **7.** Let $\epsilon_{1},\epsilon_{2},\ldots$ and $\lambda_{1},\lambda_{2},\ldots$ be sequences of positive real numbers. For each n∈N, there exists a measurable function ${\hat{\theta }}_{n}$ from (Ω,F) to $\left(\Theta_{n},B\left(\Theta_{n}\right)\right)$ that satisfies ${\hat{\theta }}_{n}\left(\omega \right)\in {\hat{\Theta }}_{n}^{*}\left(\omega \right)$ and $p\left({\hat{\theta }}_{n}\left(\omega \right)\right)\leq \inf_{\theta \in {\hat{\Theta }}_{n}^{*}\left(\omega \right)}p\left(\theta \right)+\epsilon_{n}$ for all ω∈Ω, where

$${\hat{\Theta }}_{n}^{*}\left(\omega \right)=\left\{\theta \in \Theta_{n}:M_{n}\left(\omega ,\theta \right)\leq \underset{\vartheta \in \Theta_{n}}{\inf}M_{n}\left(\omega ,\vartheta \right)+\lambda_{n}\right\}.$$

**Remark** **9.** *The mathematical content of Proposition 7 is the existence of a random function ${\hat{\theta }}_{n}$ satisfying the stated conditions. The proof applies the Sainte-Beauve measurable selection theorem (see Corollary 5.3.2 in [27]) and Theorem 2.17 of Stinchcombe and White [28], which concerns the measurability of the suprema of random functions over random sets. Proposition 7 also serves to define our estimated replicating function ${\hat{\theta }}_{n}$. That is, we take ${\hat{\theta }}_{n}$ to be any random function satisfying the conditions given in Proposition 7.*

**Remark** **10.** The random set ${\hat{\Theta }}_{n}^{*}$ can be viewed as our estimate of the set of replicators $\Theta^{*}$. It consists of all those functions $\theta \in \Theta_{n}$ such that $M_{n}\left(\theta \right)$ comes close to achieving its infimum over $\Theta_{n}$. Note that this infimum is not necessarily achieved by any $\theta \in \Theta_{n}$. The tuning parameter $\lambda_{n}$ governs how close $M_{n}\left(\theta \right)$ must be to $\inf_{\vartheta \in \Theta_{n}}M_{n}\left(\vartheta \right)$ before θ is admitted into the set ${\hat{\Theta }}_{n}^{*}$. We will require that $\lambda_{n}\to 0$ as n→∞, but at a rate that is not too fast. ${\hat{\theta }}_{n}$ is chosen such that ${\hat{\theta }}_{n}\left(\omega \right)\in {\hat{\Theta }}_{n}^{*}\left(\omega \right)$ for each ω∈Ω. Thus, if ${\hat{\Theta }}_{n}^{*}$ is an effective estimator of $\Theta^{*}$, we can expect ${\hat{\theta }}_{n}$ to come close to achieving distributional replication.

**Remark** **11.** The sequence $\epsilon_{1},\epsilon_{2},\ldots$ should be thought of as converging to zero very quickly. We would like to choose ${\hat{\theta }}_{n}$ such that $p({\hat{\theta }}_{n}\left(\omega \right))$ is equal to the infimum of p over ${\hat{\Theta }}_{n}^{*}\left(\omega \right)$ for each ω∈Ω, but in general this is not possible because the set ${\hat{\Theta }}_{n}^{*}\left(\omega \right)$ need not be compact. So instead, we choose ${\hat{\theta }}_{n}$ such that $p({\hat{\theta }}_{n}\left(\omega \right))$ is very close to the infimum of p over ${\hat{\Theta }}_{n}^{*}\left(\omega \right)$, with arbitrarily small approximation error $\epsilon_{n}$. This technical argument relates closely to what Chen [29] (p. 5561) refers to as an approximate sieve extremum estimate. Though $\lambda_{n}$ and $\epsilon_{n}$ appear to play similar roles in Proposition 7, from a more substantive perspective we wish $\epsilon_{n}$ to be as small as possible, while $\lambda_{n}$ plays a more involved role in the asymptotic results to follow, and must be chosen to converge to zero at a suitable rate.

**Remark** **12.** *If there is no relevant notion of “price” over the space of functions* Θ, *we may simply take p to be constant over* Θ. In this case, the sequence $\epsilon_{1},\epsilon_{2},\ldots$ and the function p play no role in Proposition 7. Instead, Proposition 7 merely asserts the existence of a measurable function ${\hat{\theta }}_{n}$ from (Ω,F) to $\left(\Theta_{n},B\left(\Theta_{n}\right)\right)$ that satisfies ${\hat{\theta }}_{n}\left(\omega \right)\in {\hat{\Theta }}_{n}^{*}\left(\omega \right)$ for each ω∈Ω.

It remains to show that our estimator ${\hat{\theta }}_{n}$ has desirable asymptotic properties. To ensure that ${\hat{\theta }}_{n}$ is well-behaved, the rate at which the sieve space $\Theta_{n}$ expands, and at which the tuning parameter $\lambda_{n}$ decays, must be suitably controlled. The following assumption provides a sufficient condition of this kind.

**Assumption** **5.** As n→∞, we have $\lambda_{n}\to 0$, $n^{-1}\lambda_{n}^{-2}V\left(\Theta_{n}\right)\to 0$ and $\lambda_{n}^{-1}\inf_{\theta \in \Theta_{n}}d\left(\theta ,\theta^{†}\right)\to 0$ for each $\theta^{†}\in \Theta^{†}$, where $\Theta^{†}$ is some dense subset of $\Theta^{*}$ under d.

**Remark** **13.** *The requirement that $n^{-1}\lambda_{n}^{-2}V\left(\Theta_{n}\right)\to 0$ and $\lambda_{n}^{-1}\inf_{\theta \in \Theta_{n}}d\left(\theta ,\theta^{†}\right)\to 0$ for each $\theta^{†}\in \Theta^{†}$ places opposing constraints on the rate of expansion of $\Theta_{n}$ as n→∞. The complexity of $\Theta_{n}$ must increase sufficiently fast for the sieve approximation error $\inf_{\theta \in \Theta_{n}}d\left(\theta ,\theta^{†}\right)$ to tend to zero faster than $\lambda_{n}$ for each $\theta^{†}\in \Theta^{†}$, but not so fast that $V(\Theta_{n})$ increases faster than $n\lambda_{n}^{2}$. On the other hand, the rate of decay of $\lambda_{n}$ may be arbitrarily slow, provided that $\lambda_{n}\to 0$.*

Our final result of this section indicates that, when the above assumptions are satisfied, in large samples we can expect our estimated function to be close to achieving distributional replication, and close to achieving the minimum cost among replicators. We first require some additional notation. Let $P^{{\hat{\theta }}_{n}}$ be the probability measure on $B(\Theta_{n})$ given by $P^{{\hat{\theta }}_{n}}B=P{\hat{\theta }}_{n}^{-1}B$ for each $B\in B(\Theta_{n})$, and let $P^{{\hat{\theta }}_{n}\left(X\right)}$ be the probability measure on B(R) given by $P^{{\hat{\theta }}_{n}\left(X\right)}B=P^{{\hat{\theta }}_{n}}⊗P^{X}\left\{\left(\theta ,x\right)\in \Theta_{n}\times R^{X}:\theta \left(x\right)\in B\right\}$ for each B∈B(R). Note that for $P^{{\hat{\theta }}_{n}\left(X\right)}$ to be well defined we need $B(\Theta_{n})$ to be an admissible structure for $\Theta_{n}$; this condition was given in Assumption 3. We can think of $P^{{\hat{\theta }}_{n}\left(X\right)}$ as the probability distribution of ${\hat{\theta }}_{n}\left(X\right)$ when ${\hat{\theta }}_{n}$ and *X* are distributed independently of one another.

**Proposition** **8.** As n→∞, $P^{{\hat{\theta }}_{n}\left(X\right)}\Rightarrow P^{Y}$ and $P\{\omega :p\left({\hat{\theta }}_{n}\left(\omega \right)\right)>\inf_{\theta \in \Theta^{*}}p\left(\theta \right)+\varepsilon \}\to 0$ for any ε>0.

**Remark** **14.** Proposition 8 indicates that ${\hat{\theta }}_{n}$ can be expected to perform well with respect to the dual goals of distributional replication and cost minimization in large samples. This duality complicates any discussion of the optimal selection of the tuning parameter $\lambda_{n}$. When $\lambda_{n}$ is large, we include functions in our estimated set ${\hat{\Theta }}_{n}^{*}$ for which the empirical evidence for distributional replication is weaker, but we also minimize the function p over a larger set. In applications, the best choice of $\lambda_{n}$ would depend on an individual’s relative preference for distributional replication, quantified by M(θ), and cost minimization, quantified by p(θ).

## 4. Distributional Replication Using Options

In this section we consider the problem of choosing a portfolio of options on some financial asset such that the payoff from our portfolio after a specified period of time has approximately the same statistical distribution as the payoff from a $1 investment in some other asset over the same time period. We would like to find the cheapest portfolio of options such that distributional replication is achieved; in particular, we would like the cost of the portfolio to be $1 or less. We will show how this problem of portfolio selection can be interpreted and solved using the machinery developed in the previous two sections.

We suppose that the random variables *X* and *Y* represent the dollar denominated payoffs after one period from a $1 investment in each of two assets. The asset with payoff *X* will be referred to as the base asset, and the asset with payoff *Y* will be referred to as the target asset. The price of a one share investment in either asset is taken to be $1. We assume that *X* and *Y* are nonnegative and may be arbitrarily large with nonzero probability, so that $R^{X}=R^{Y}=\left[0,\infty \right)$ under Assumption 1. We may thus replace Assumption 1 with the following more restrictive condition.

**Assumption** **6.** *$F^{X}$ and $F^{Y}$ are continuous and strictly increasing on [0,∞), and zero on (−∞,0].*

We find the payoff distribution of the target asset to be desirable, but we seek to achieve this distribution by investing in a portfolio composed of the base asset itself and a basket of European put and call options written on the base asset, with the options expiring after one period. The payoff of such a portfolio after one period is a nonrandom function of *X*; for instance, the payoff from a European call option with strike price *s* after one period is given by max{0,X−s}, while the payoff from a European put option with strike price *s* after one period is given by max{0,s−X}. We also allow our portfolio to include an investment in risk-free zero-coupon bonds with $1 par value, expiring after one period. The payoff from such a bond after one period is simply $1. We allow our portfolio to include long or short positions in each of the component assets, but the payoff from the complete portfolio must be nonnegative.

The payoff from a portfolio of options and bonds after one period is a nonrandom function of *X*. Thus, we can think of a portfolio as a function θ∈Θ, and write the payoff from the portfolio as θ(X). Suppose our portfolio includes options at *m* different strike prices $s_{1},\ldots ,s_{m}$, with $0<s_{1}<\cdots <s_{m}<\infty$. Without loss of generality, we may consider all options to be call options, since the payoff function for a put option with strike price $s_{i}$ can be replicated by selling one share of the base asset, purchasing a call option with strike price $s_{i}$, and purchasing $s_{i}$ zero-coupon bonds. Suppose we form a portfolio by purchasing $\beta_{1}$ bonds, $\beta_{2}$ shares in the base asset, and $\beta_{i+2}$ call options at strike price $s_{i}$, with i=1,…,m. The payoff function corresponding to our portfolio is then given by

$$\theta \left(x;\beta ,s\right)=\beta_{1}+\beta_{2}x+\sum_{i=1}^{m}\beta_{i+2}\max\left\{0,x-s_{i}\right\},$$

where x∈[0,∞). For fixed $s=(s_{1},\ldots ,s_{m})$, the collection of functions $\left\{\theta \left(\cdot ;\beta ,s\right):\beta \in R^{m+2}\right\}$ consists of all the continuous functions from [0,∞) to R that are linear on each of the m+1 subintervals $\left(0,s_{1}\right),\left(s_{1},s_{2}\right),\ldots ,\left(s_{m},\infty \right)$. To ensure that the payoff from our portfolio is nonnegative, we require that β lies in a suitable subset of $R^{m+2}$. We will let $\Psi_{m}\left(s\right)$ denote the collection of all continuous functions from [0,∞) to [0,∞) that are linear on each of the m+1 subintervals $\left(0,s_{1}\right),\left(s_{1},s_{2}\right),\ldots ,\left(s_{m},\infty \right)$, and let $B(\Psi_{m}\left(s\right))$ denote the Borel σ-field on $\Psi_{m}\left(s\right)$ generated by *d*.

**Proposition** **9.** *For fixed $s\in R^{m}$ with $0<s_{1}<\cdots <s_{m}$, we have (i) $V(\Psi_{m}\left(s\right))=m+3$; (ii) $\left(\Psi_{m}\left(s\right),d\right)$ is a Polish metric space; and (iii) $B(\Psi_{m}\left(s\right))$ is an admissible structure for $\Psi_{m}\left(s\right)$.*

We can see from Proposition 9 and Remark 7 that $\Psi_{m}\left(s\right)$ satisfies the conditions placed on $\Theta_{n}$ in Assumption 3. The main idea behind the application discussed in this section is that $\Psi_{m}\left(s\right)$, the space of nonnegative payoff functions achievable using strike prices *s*, can be used to play the role of the sieve space $\Theta_{n}$ described in the previous section. We obtain an expanding sequence of sieve spaces by assuming that the collection of strike prices *s* varies with the sample size *n*, becoming more dense (in a sense soon to be made precise) as *n* increases. Suppose that $m_{1},m_{2},\ldots$ is a nondecreasing sequence of natural numbers with $m_{n}\to \infty$ and $m_{n}/n\to 0$ as n→∞. Let $\left\{s_{i,n}:i=1,\ldots ,m_{n};n\in N\right\}$ be a triangular array of positive real numbers satisfying (i) $0<s_{1,n}<\cdots <s_{m_{n},n}$ for each n∈N, and (ii) $\left\{s_{1,n},\ldots ,s_{m_{n},n}\right\}\subseteq \left\{s_{1,n+1},\ldots ,s_{m_{n+1},n+1}\right\}$ for each n∈N. We define our expanding sequence of sieve spaces by setting $\Theta_{n}=\Psi_{m_{n}}\left(s_{1,n},\ldots ,s_{m_{n},n}\right)$. Proposition 9 implies that this choice of $\Theta_{n}$ satisfies Assumption 3, with $V\left(\Theta_{n}\right)=m_{n}+3$.

In the context of the present application, the function *p* introduced in the previous section describes, literally, the price of each payoff function θ∈Θ. For a payoff function $\theta \in \Theta_{n}$, we can calculate the price p(θ) directly from the prices of bonds and options. Consider the function $\theta \left(x;\beta ,s\right)=\beta_{1}+\beta_{2}x+\sum_{i=1}^{m}\beta_{i+2}\max\left\{0,x-s_{i}\right\}$ defined earlier. Let $p_{1}$ denote the price of a bond, $p_{2}$ denote the price of a share in the base asset, and $p_{i+2}$ denote the price of a call option with strike price $s_{i}$, where i=1,…,m. Note that $p_{2}=1$ by assumption. The price of θ(·;β,s) is simply $\sum_{i=1}^{m+2}p_{i}\beta_{i}$. In this way we can calculate p(θ) for any $\theta \in \Theta_{n}$, provided we observe the bond price $p_{1}$ and the prices of call options at strike prices $s_{1,n},\ldots ,s_{m_{n},n}$.

Assumption 5 imposes a condition on the rate of decay of the sieve approximation error: we require that $\lambda_{n}^{-1}\inf_{\theta \in \Theta_{n}}d\left(\theta ,\theta^{†}\right)\to 0$ for each $\theta^{†}\in \Theta^{†}$, where $\Theta^{†}$ is some dense subset of $\Theta^{*}$ under *d*. The following result shows how $\Theta^{†}$ may be chosen such that this condition is satisfied when our sieve space corresponds to portfolios of options.

**Proposition** **10.** Let $\Theta^{†}$ denote the set of all functions $\theta^{†}\in \Theta^{*}$ such that $F^{Y}\circ \theta^{†}\circ Q^{X}$ is Lipschitz continuous. Then $\Theta^{†}$ is dense in $\Theta^{*}$ under d. Also, when $\Theta_{n}=\Psi_{m_{n}}\left(s_{1,n},\ldots ,s_{m_{n},n}\right)$, as n→∞ we have

$$\underset{\theta \in \Theta_{n}}{\inf}d\left(\theta ,\theta^{†}\right)=O\left(m_{n}\underset{0\leq i\leq m_{n}}{\sup}P^{X}{\left(s_{i,n},s_{i+1,n}\right)}^{2}\right)$$

*for each $\theta^{†}\in \Theta^{†}$, where $s_{0,n}=0$ and $s_{m_{n}+1,n}=\infty$.*

Proposition 10 reveals that our sequence of sieve spaces constructed using option payoffs can approximate replicating functions satisfying a deformed Lipschitz condition, provided that $\sup_{0\leq i\leq m_{n}}P^{X}\left(s_{i,n},s_{i+1,n}\right)$ decays to zero at a suitable rate. Further, that set of deformed Lipschitz continuous replicating functions is dense in the set of all replicating functions. If we could choose our strike prices such that $P^{X}\left(s_{i,n},s_{i+1,n}\right)$ was constant across $i=0,\ldots ,m_{n}$, we would have $\inf_{\theta \in \Theta_{n}}d\left(\theta ,\theta^{†}\right)=O\left(m_{n}^{-1}\right)$ for each $\theta^{†}\in \Theta^{†}$.

Proposition 10 and part (i) of Proposition 9 show how the choice of strike prices is constrained by Assumption 5. Specifically, the conditions on $\Theta_{n}$ imposed by Assumption 5 may be rewritten as follows: $n^{-1}\lambda_{n}^{-2}m_{n}\to 0$ and $\lambda_{n}^{-1}m_{n}\sup_{0\leq i\leq m_{n}}P^{X}{\left(s_{i,n},s_{i+1,n}\right)}^{2}\to 0$ as n→∞. If our strike prices are chosen such that $P^{X}\left(s_{i,n},s_{i+1,n}\right)$ is constant across $i=0,\ldots ,m_{n}$, Assumption 5 will be satisfied provided that $\lambda_{n}=o\left(1\right)$, $m_{n}=o\left(n\lambda_{n}^{2}\right)$ and $m_{n}^{-1}=o\left(\lambda_{n}\right)$. For instance, we could choose $m_{n}∼n^{a}$ and $\lambda_{n}∼n^{-b}$, with 0<b<a<1−2b. As noted in Remark 14, it is difficult to see how an optimal choice of $m_{n}$ and $\lambda_{n}$ could be made in practice, because the two parameters may have different effects on the twin criterion functions M(θ) and p(θ), and one’s relative preference for optimizing with respect to those two functions may be idiosyncratic. It is perhaps best to experiment with a range of different values for $m_{n}$ and $\lambda_{n}$. Further, the choice of strike prices is likely to be constrained by the strike prices being actively traded on the market.

## 5. Conclusions

In this paper we have developed a mathematical framework for thinking about the estimation of a function θ such that θ(X) has the same distribution as *Y*. We have discussed the relevance of our results to financial applications in which one seeks to find the cheapest way to achieve a desired payoff distribution by trading liquid assets. We now briefly discuss two possible extensions of our results that may prove fruitful.

In terms of the relevance of our technical conditions in financial applications, the elephant in the room is clearly Assumption 2, which imposes an iid condition on the random variables $\left(X_{i},Y_{i}\right)$, i=1,…,n. It is certainly the case that time series of financial returns typically do not behave as though they were distributed independently over time, as is clear from the voluminous literature on stochastic volatility. The iid condition comes into play in the proof of Proposition 6, in which results in empirical process theory are used to establish a uniform bound on the error in the approximation of *M* by $M_{n}$ over our sieve space $\Theta_{n}$. The results we apply are based on iid conditions, but generalizations suitable for dependent data are available [31,32,33]. It seems likely that, with some strengthening of the rate conditions in Assumption 5, the results in this paper could be adapted to allow for dependent data. However, by allowing for the possibility of serial dependence, a further question is raised. The methods we have proposed are designed such that the unconditional distribution of θ(X) is approximately equal to the unconditional distribution of *Y*. If data are serially dependent, the more relevant objects may be distributions that are conditional on past information. Though we acknowledge the importance of this issue, it goes beyond the scope of this paper.

A second potential extension of our results would be to consider the replication of multivariate distributions. As discussed in the introduction, Kat and Palaro [7,8] consider estimating a transformation θ of a pair of random variables *X* and *Z* such that *X* and θ(X,Z) have the same joint distribution as *X* and *Y*. The difficulty with adapting our own method to this approach is that the class of bivariate functions that can be approximated by portfolios of options written on individual assets is rather small. One possible solution would be to consider portfolios formed from derivative securities that are written on multiple underlying assets; another would be to forgo exact distributional replication, and seek the closest distributional match from a smaller class of multivariate payoff functions that is approximable using portfolios of simple options. We leave these possibilities for future research.

## Data Availability

Data is contained within the article.

## Appendix A. Proofs

**Proof** **of** **Proposition** **1.** Choose a point c∈(0,1), and let ${\tilde{\theta }}^{c}:\left[0,1\right]\to \left(0,1\right)$ be defined by ${\tilde{\theta }}^{c}\left(u\right)=u/c$ for u∈(0,c), ${\tilde{\theta }}^{c}\left(u\right)=\left(u-c\right)/\left(1-c\right)$ for u∈(c,1), and ${\tilde{\theta }}^{c}\left(u\right)=\frac{1}{2}$ for u∈{0,c,1}. Let $\theta^{c}:R^{X}\to R^{Y}$ be defined by $\theta^{c}\left(x\right)=Q^{Y}\circ {\tilde{\theta }}^{c}\circ F^{X}\left(x\right)$. $F^{X}$ is continuous under Assumption 1, so $F^{X}\left(X\right)∼U\left(0,1\right)$. Hence, for any a∈(0,1), we have

$$\begin{array}{cc} P^{X}\left\{x:{\tilde{\theta }}^{c}\circ F^{X}\left(x\right)\leq a\right\} & =\int_{0}^{1}1\left\{{\tilde{\theta }}^{c}\left(u\right)\leq a\right\}du\\ & =\int_{0}^{1}1\left\{u\leq ac\;\mathrm{or}\;c\leq u\leq c+a-ac\right\}du=a, \end{array}$$

implying that ${\tilde{\theta }}^{c}\circ F^{X}\left(X\right)∼U\left(0,1\right)$. Therefore, $\theta^{c}\left(X\right)∼Y$, and so $\theta^{c}$ is a replicating function for *X* and *Y*. If we choose $c_{0},c_{1}\in \left(0,1\right)$ with $c_{0}\neq c_{1}$, then ${\tilde{\theta }}^{c_{0}}\left(u\right)\neq {\tilde{\theta }}^{c_{1}}\left(u\right)$ for $u\notin \{0,c_{0},c_{1},1\}$. Since $Q^{Y}$ is strictly increasing under Assumption 1, it follows that $\theta^{c_{0}}\left(x\right)\neq \theta^{c_{1}}\left(x\right)$ for $x\notin \{Q^{X}\left(0\right),Q^{X}\left(c_{0}\right),Q^{X}\left(c_{1}\right),Q^{X}\left(1\right)\}$, and so continuity of $F^{X}$ implies that $\theta^{c_{0}}\left(x\right)\neq \theta^{c_{1}}\left(x\right)$ for all *x* in a set of $P^{X}$-measure one. Thus, by allowing *c* to vary over (0,1), we obtain an uncountable collection of replicating functions, no two of which are equal on a set of positive $P^{X}$-measure. □

**Proof** **of** **Proposition** **2.** For $\theta_{0},\theta_{1}\in \Theta$ we can use the triangle inequality to show that

$$\left|M\left(\theta_{1}\right)-M\left(\theta_{0}\right)\right|\leq \int_{R^{Y}}\left|F^{X}\left(y;\theta_{1}\right)-F^{X}\left(y;\theta_{0}\right)\right|dF^{Y}\left(y\right).$$

For each $y\in R^{Y}$ we have

$$\begin{array}{ccc} |F^{X}\left(y;\theta_{1}\right)-F^{X}\left(y;\theta_{0}\right)| & = & |P^{X}\left\{x:\theta_{1}\left(x\right)\leq y\right\}-P^{X}\left\{x:\theta_{0}\left(x\right)\leq y\right\}|\\ & = & |P^{X}\left\{x:\theta_{1}\left(x\right)\leq y<\theta_{0}\left(x\right)\right\}-P^{X}\left\{x:\theta_{0}\left(x\right)\leq y<\theta_{1}\left(x\right)\right\}|, \end{array}$$

and so applying the triangle inequality again we obtain

$$\begin{array}{cc} \left|M\left(\theta_{1}\right)-M\left(\theta_{0}\right)\right| & \leq \int_{R^{Y}}P^{X}\left\{x:\theta_{1}\left(x\right)\leq y<\theta_{0}\left(x\right)\right\}dF^{Y}\left(y\right)\\ & +\int_{R^{Y}}P^{X}\left\{x:\theta_{0}\left(x\right)\leq y<\theta_{1}\left(x\right)\right\}dF^{Y}\left(y\right). \end{array}$$

Tonelli’s theorem implies that

$$\int_{R^{Y}}P^{X}\left\{x:\theta_{1}\left(x\right)\leq y<\theta_{0}\left(x\right)\right\}dF^{Y}\left(y\right)=\int_{R^{X}}\int_{R^{Y}}1\left\{\theta_{1}\left(x\right)\leq y<\theta_{0}\left(x\right)\right\}dF^{Y}\left(y\right)dF^{X}\left(x\right).$$

The distribution function $F^{Y}$ is continuous under Assumption 1, and so we have

$$\int_{R^{Y}}1\left\{\theta_{1}\left(x\right)\leq y<\theta_{0}\left(x\right)\right\}dF^{Y}\left(y\right)=\max\left\{F^{Y}\left(\theta_{0}\left(x\right)\right)-F^{Y}\left(\theta_{1}\left(x\right)\right),0\right\}$$

for each $x\in R^{X}$. Therefore,

$$\int_{R^{Y}}P^{X}\left\{x:\theta_{1}\left(x\right)\leq y<\theta_{0}\left(x\right)\right\}dF^{Y}\left(y\right)=\int_{R^{X}}\max\left\{F^{Y}\left(\theta_{0}\left(x\right)\right)-F^{Y}\left(\theta_{1}\left(x\right)\right),0\right\}dF^{X}\left(x\right).$$

Similarly, we have

$$\int_{R^{Y}}P^{X}\left\{x:\theta_{0}\left(x\right)\leq y<\theta_{1}\left(x\right)\right\}dF^{Y}\left(y\right)=\int_{R^{X}}\max\left\{F^{Y}\left(\theta_{1}\left(x\right)\right)-F^{Y}\left(\theta_{0}\left(x\right)\right),0\right\}dF^{X}\left(x\right),$$

and so

$$\begin{array}{c} |M\left(\theta_{1}\right)-M\left(\theta_{0}\right)|\leq \int_{R^{X}}\left|F^{Y}\left(\theta_{1}\left(x\right)\right)-F^{Y}\left(\theta_{0}\left(x\right)\right)\right|dF^{X}\left(x\right)=d\left(\theta_{0},\theta_{1}\right). \end{array}$$

 □

**Proof** **of** **Proposition** **3.** It is obvious that M(θ)=0 if $\theta \in \Theta^{*}$. We will prove the reverse implication. Suppose M(θ)=0. Then $F^{X}\left(y;\theta \right)=F^{Y}\left(y\right)$ for all *y* in a set of $P^{Y}$-measure one. Suppose $F^{X}\left(c_{0};\theta \right)\neq F^{Y}\left(c_{0}\right)$ for some $c_{0}$ in the interior of $R^{Y}$. Right continuity of $F^{X}\left(\cdot ;\theta \right)$ and $F^{Y}$ ensures that $F^{X}\left(y;\theta \right)\neq F^{Y}\left(y\right)$ for all *y* in some open interval $\left(c_{0},c_{1}\right)$. Since $F^{Y}$ is strictly increasing on $R^{Y}$ under Assumption 1, $\left(c_{0},c_{1}\right)$ must have strictly positive $P^{Y}$-measure, leading to a contradiction. Thus it must be the case that $F^{X}\left(y;\theta \right)=F^{Y}\left(y\right)$ for all *y* in the interior of $R^{Y}$. Since $F^{X}\left(\cdot ;\theta \right)$ is nondecreasing and takes values between zero and one, and $F^{Y}$ increases continuously from zero to one over $R^{Y}$, it follows that $F^{X}\left(y;\theta \right)=F^{Y}\left(y\right)$ for all y∈R, so that $\theta \in \Theta^{*}$. □

**Proof** **of** **Proposition** **4.** If $F^{X}\left(y;\theta_{n}\right)\to F^{Y}\left(y\right)$ for each y∈R, then $M(\theta_{n})\to 0$ by dominated convergence. Suppose $F^{X}\left(c_{0};\theta_{n}\right)↛F^{Y}\left(c_{0}\right)$ for some $c_{0}\in R$. Then there must be an increasing sequence of natural numbers $n_{1},n_{2},\ldots$ and a real number ε>0 (or ε<0) such that $F^{X}\left(c_{0};\theta_{n_{k}}\right)\geq F^{Y}\left(c_{0}\right)+\varepsilon$ (resp. $F^{X}\left(c_{0};\theta_{n_{k}}\right)\leq F^{Y}\left(c_{0}\right)+\varepsilon$) for all *k* sufficiently large. Suppose ε>0. Since $F^{Y}$ is continuous under Assumption 1, we may choose $c_{1}>c_{0}$ such that $F^{Y}\left(c_{1}\right)=F^{Y}\left(c_{0}\right)+\varepsilon /2$. Monotonicity of $F^{X}\left(\cdot ;\theta_{n_{k}}\right)$ and $F^{Y}$ then ensures that $F^{X}\left(y;\theta_{n_{k}}\right)\geq F^{Y}\left(y\right)+\varepsilon /2$ for all $y\in [c_{0},c_{1}]$, for all *k* sufficiently large. Consequently, we have

$$\int |F^{X}\left(y;\theta_{n_{k}}\right)-F^{Y}\left(y\right)|dF^{Y}\left(y\right)\geq \frac{\varepsilon }{2}\int_{c_{0}}^{c_{1}}dF^{Y}\left(y\right)=\frac{\varepsilon }{2}\left(F^{Y}\left(c_{1}\right)-F^{Y}\left(c_{0}\right)\right)=\frac{\varepsilon^{2}}{4}>0$$

for all *k* sufficiently large, implying that $M(\theta_{n})↛0$. □

**Proof** **of** **Proposition** **5.** Since (θ,x)↦θ(x) is $T_{n}⊗B\left(R^{X}\right)$-measurable, it follows that (θ,x,y)↦1{θ(x)≤y} is $T_{n}⊗B\left(R^{X}\right)⊗B\left(R\right)$-measurable. Tonelli’s theorem thus implies that $\left(\theta ,y\right)\mapsto \int_{R^{X}}1\left\{\theta \left(x\right)\leq y\right\}dP^{X}\left(x\right)=F^{X}\left(y;\theta \right)$ is $T_{n}⊗B\left(R\right)$-measurable, justifying the following interchange of integrals:

$$\begin{array}{cc} \int_{\Theta_{n}}MdP^{\theta_{n}} & =\int_{\Theta_{n}}\int_{R^{Y}}\left|F^{X}\left(y;\theta \right)-F^{Y}\left(y\right)\right|dF^{Y}\left(y\right)dP^{\theta_{n}}\left(\theta \right)\\ \; & =\int_{R^{Y}}\int_{\Theta_{n}}\left|F^{X}\left(y;\theta \right)-F^{Y}\left(y\right)\right|dP^{\theta_{n}}\left(\theta \right)dF^{Y}\left(y\right). \end{array}$$

We thus have

$$\int_{\Theta_{n}}MdP^{\theta_{n}}\geq \int_{R^{Y}}\left|\int_{\Theta_{n}}F^{X}\left(y;\theta \right)dP^{\theta_{n}}\left(\theta \right)-F^{Y}\left(y\right)\right|dF^{Y}\left(y\right).$$

Again using the $T_{n}⊗B\left(R\right)$-measurability of $\left(\theta ,y\right)\mapsto F^{X}\left(y;\theta \right)$, Tonelli’s theorem implies that

$$\int_{\Theta_{n}}F^{X}\left(y;\theta \right)dP^{\theta_{n}}\left(\theta \right)=\int_{\Theta_{n}}\int_{R^{X}}1\left\{\theta \left(x\right)\leq y\right\}dP^{X}\left(x\right)dP^{\theta_{n}}\left(\theta \right)=P^{\theta_{n}\left(X\right)}\left(-\infty ,y\right]$$

for each y∈R. Letting $F^{\theta_{n}\left(X\right)}$ denote the cdf corresponding to $P^{\theta_{n}\left(X\right)}$, we now have

$$\int_{\Theta_{n}}MdP^{\theta_{n}}\geq \int_{R^{Y}}\left|F^{\theta_{n}\left(X\right)}\left(y\right)-F^{Y}\left(y\right)\right|dF^{Y}\left(y\right).$$

Arguing as in the proof of Proposition 4, we can show that $\int_{R^{Y}}\left|F^{\theta_{n}\left(X\right)}\left(y\right)-F^{Y}\left(y\right)\right|dF^{Y}\left(y\right)\to 0$ if and only if $F^{\theta_{n}\left(X\right)}\left(y\right)\to F^{Y}\left(y\right)$ for each y∈R. Thus, $\int_{\Theta_{n}}MdP^{\theta_{n}}\to 0$ implies $F^{\theta_{n}\left(X\right)}\to F^{Y}$ pointwise, which is equivalent to $P^{\theta_{n}\left(X\right)}\Rightarrow P^{Y}$. □

**Proof** **of** **Proposition** **6.** Elementary arguments can be used to show that, for all θ∈Θ,

(A1) $$\begin{array}{cc} |M_{n}\left(\theta \right)-M\left(\theta \right)| & \leq \int |F_{n}^{Y}\left(y\right)-F^{Y}\left(y\right)|dF_{n}^{Y}\left(y\right)+\int \left|F_{n}^{X}\left(y;\theta \right)-F^{X}\left(y;\theta \right)\right|dF_{n}^{Y}\left(y\right)\\ \; & +\left|\int |F^{X}\left(y;\theta \right)-F^{Y}\left(y\right)|dF_{n}^{Y}\left(y\right)-\int \left|F^{X}\left(y;\theta \right)-F^{Y}\left(y\right)\right|dF^{Y}\left(y\right)\right|. \end{array}$$

We will establish a uniform bound on the order of each of the three terms on the right-hand side of (A1). These bounds will be expressed in terms of the outer expectation operator $E^{*}$, denoting outer integration of nonnegative functions defined on the underlying probability space (Ω,F,P); see e.g., Section 1.2 in [30]. Obtaining a bound for the first term is simple as it does not depend on θ: Donsker’s theorem yields

$$E^{*}\int |F_{n}^{Y}\left(y\right)-F^{Y}\left(y\right)|dF_{n}^{Y}\left(y\right)\leq E^{*}\underset{y\in R}{\sup}\left|F_{n}^{Y}\left(y\right)-F^{Y}\left(y\right)\right|=O\left(n^{-1/2}\right).$$

For the second term on the right-hand side of (A1), we have

$$\begin{array}{cc} E^{*}\underset{\theta \in \Theta_{n}}{\sup}\int \left|F_{n}^{X}\left(y;\theta \right)-F^{X}\left(y;\theta \right)\right|dF_{n}^{Y}\left(y\right) & \leq E^{*}\underset{\theta \in \Theta_{n}}{\sup}\underset{y\in R}{\sup}\left|F_{n}^{X}\left(y;\theta \right)-F^{X}\left(y;\theta \right)\right|\\ & =E^{*}\underset{g\in G_{n}}{\sup}\left|P_{n}^{X}g-P^{X}g\right|, \end{array}$$

where $G_{n}$ is the class of indicator functions of sets of the form $\left\{x\in R^{X}:\theta \left(x\right)\leq y\right\}$ with $\theta \in \Theta_{n}$ and y∈R. Note that $G_{n}$ is the collection of indicators of all complements of sets majorized by $\Theta_{n}$. Since $\Theta_{n}$ is VC-major with dimension $V(\Theta_{n})$, $G_{n}$ must be VC-subgraph with dimension $V(\Theta_{n})$. Hence Theorem 2.6.7 in [30] implies that, for any ε∈(0,1) and any probability measure *Q* on B(R), there exists K<∞ such that we have the uniform entropy bound $N\left(\varepsilon ,G_{n},L_{2}\left(Q\right)\right)\leq KV\left(\Theta_{n}\right){\left(16e\right)}^{V(\Theta_{n})}\varepsilon^{-2(V\left(\Theta_{n}\right)-1)}$. Theorem 2.14.1 in [30] thus gives $E^{*}\sup_{g\in G_{n}}\left|P_{n}^{X}g-P^{X}g\right|=O\left(\sqrt{V(\Theta_{n})/n}\right)$, implying that

$$E^{*}\underset{\theta \in \Theta_{n}}{\sup}\int \left|F_{n}^{X}\left(y;\theta \right)-F^{X}\left(y;\theta \right)\right|dF_{n}^{Y}\left(y\right)=O\left(\sqrt{V(\Theta_{n})/n}\right).$$

For the third term on the right-hand side of (A1), we have

$$\begin{array}{cc} \; & E^{*}\underset{\theta \in \Theta_{n}}{\sup}\left|\int |F^{X}\left(y;\theta \right)-F^{Y}\left(y\right)|dF_{n}^{Y}\left(y\right)-\int \left|F^{X}\left(y;\theta \right)-F^{Y}\left(y\right)\right|dF^{Y}\left(y\right)\right|\\ & =E^{*}\underset{h\in H_{n}}{\sup}\left|P_{n}^{Y}h-P^{Y}h\right|, \end{array}$$

where $H_{n}$ is the class of functions $\left\{|F^{X}\left(\cdot ;\theta \right)-F^{Y}\left(\cdot \right)|:\theta \in \Theta_{n}\right\}$. Consider the simpler class of functions $H_{n}^{0}=\left\{F^{X}\left(\cdot ;\theta \right):\theta \in \Theta_{n}\right\}$. Since $H_{n}^{0}$ is a subset of the collection of monotone increasing functions from R to [0,1], Theorem 2.7.5 in [30] implies the existence of K<∞ such that we have the uniform bracketing entropy bound $N_{\left[\right]}\left(\varepsilon ,H_{n}^{0},L_{2}\left(P^{Y}\right)\right)\leq K\varepsilon^{-1}$ for all ε∈(0,1). It is straightforward to show that $N_{\left[\right]}\left(\varepsilon ,H_{n},L_{2}\left(P^{Y}\right)\right)\leq N_{\left[\right]}\left(\varepsilon ,H_{n}^{0},L_{2}\left(P^{Y}\right)\right)$, and so Theorem 2.14.2 in [30] gives $E^{*}\sup_{h\in H_{n}}\left|P_{n}^{Y}h-P^{Y}h\right|=O\left(n^{-1/2}\right)$, implying that

$$E^{*}\underset{\theta \in \Theta_{n}}{\sup}\left|\int |F^{X}\left(y;\theta \right)-F^{Y}\left(y\right)|dF_{n}^{Y}\left(y\right)-\int \left|F^{X}\left(y;\theta \right)-F^{Y}\left(y\right)\right|dF^{Y}\left(y\right)\right|=O\left(n^{-1/2}\right).$$

Collecting together these bounds on the order of the terms on the right-hand side of (A1), we obtain

$$E^{*}\underset{\theta \in \Theta_{n}}{\sup}\left|M_{n}\left(\theta \right)-M\left(\theta \right)\right|=O\left(\sqrt{V(\Theta_{n})/n}\right).$$

It remains only to show that $\omega \mapsto \sup_{\theta \in \Theta_{n}}\left|M_{n}\left(\omega ,\theta \right)-M\left(\theta \right)\right|$ is F-measurable. Corollary 5.3.5 in [27] implies that $\omega \mapsto \sup_{\theta \in \Theta_{n}}\left|M_{n}\left(\omega ,\theta \right)-M\left(\theta \right)\right|$ is universally F-measurable if $\left(\Theta_{n},B\left(\Theta_{n}\right)\right)$ is a Souslin measurable space and $\left(\omega ,\theta \right)\mapsto |M_{n}\left(\omega ,\theta \right)-M\left(\theta \right)|$ is $F⊗B(\Theta_{n})$-measurable. Since (Ω,F,P) is complete under Assumption 2, universal F-measurability implies F-measurability. Assumption 3 states that $\left(\Theta_{n},B\left(\Theta_{n}\right)\right)$ is a Souslin measurable space, and θ↦M(θ) is continuous and hence $B(\Theta_{n})$-measurable by Proposition 3, so it suffices for us to show that $\left(\omega ,\theta \right)\mapsto M_{n}\left(\omega ,\theta \right)$ is $F⊗B(\Theta_{n})$-measurable. Observe that

(A2) $$M_{n}\left(\omega ,\theta \right)=\frac{1}{n}\sum_{i=1}^{n}\left|\frac{1}{n}\sum_{j=1}^{n}1\left\{\theta \left(X_{j}\left(\omega \right)\right)\leq Y_{i}\left(\omega \right)\right\}-\frac{1}{n}\sum_{j=1}^{n}1\left\{Y_{j}\left(\omega \right)\leq Y_{i}\left(\omega \right)\right\}\right|.$$

It is clear from (A2) that $\left(\omega ,\theta \right)\mapsto M_{n}\left(\omega ,\theta \right)$ is $F⊗B(\Theta_{n})$-measurable if $\left(\omega ,\theta \right)\mapsto \theta (X_{j}\left(\omega \right))$ is $F⊗B(\Theta_{n})$-measurable for any *j*. This condition is satisfied for each *j* since $B(\Theta_{n})$ is an admissible structure for $\Theta_{n}$ under Assumption 3, and each $X_{j}$ is F-measurable. □

**Proof** **of** **Proposition** **7.** We showed in the proof of Proposition 6 that $\left(\omega ,\theta \right)\mapsto M_{n}\left(\omega ,\theta \right)$ is $F⊗B(\Theta_{n})$-measurable. Since $\left(\Theta_{n},B\left(\Theta_{n}\right)\right)$ is a Souslin measurable space, it follows from Corollary 5.3.5 in [27] that $\omega \mapsto \inf_{\theta \in \Theta_{n}}M_{n}\left(\omega ,\theta \right)$ is F-measurable. Consequently, we have

$$\mathrm{gr}\left({\hat{\Theta }}_{m}^{*}\right)=\left\{\left(\omega ,\theta \right)\in \Omega \times \Theta_{n}:M_{n}\left(\omega ,\theta \right)\leq \underset{\vartheta \in \Theta_{n}}{\inf}M_{n}\left(\omega ,\vartheta \right)+\lambda_{n}\right\}\in F⊗B\left(\Theta_{n}\right),$$

where $\mathrm{gr}({\hat{\Theta }}_{m}^{*})$ denotes the graph of ${\hat{\Theta }}_{m}^{*}$. Therefore, since *p* is continuous and hence $B(\Theta_{n})$-measurable under Assumption 4, Theorem 2.17(a) of Stinchcombe and White [28] implies that $\omega \mapsto \inf_{\theta \in {\hat{\Theta }}_{m}^{*}\left(\omega \right)}p\left(\theta \right)$ is F-analytic, and hence F-measurable given that (Ω,F,P) is complete under Assumption 2. Let $H_{n}:\Omega \times \Theta_{n}\to R$ be the function defined by

$$H_{n}\left(\omega ,\theta \right)=\max\left\{M_{n}\left(\omega ,\theta \right)-\underset{\vartheta \in \Theta_{n}}{\inf}M_{n}\left(\omega ,\vartheta \right)-\lambda_{n},\;p\left(\theta \right)-\underset{\vartheta \in {\hat{\Theta }}_{n}^{*}\left(\omega \right)}{\inf}p\left(\vartheta \right)-\epsilon_{n}\right\}.$$

We have shown that $\left(\omega ,\theta \right)\mapsto M_{n}\left(\omega ,\theta \right)$ is $F⊗B(\Theta_{n})$-measurable, $\omega \mapsto \inf_{\theta \in \Theta_{n}}M_{n}\left(\omega ,\theta \right)$ is F-measurable, θ↦p(θ) is $B(\Theta_{n})$-measurable, and $\omega \mapsto \inf_{\theta \in {\hat{\Theta }}_{m}^{*}\left(\omega \right)}p\left(\theta \right)$ is F-measurable, so it must be the case that $\left(\omega ,\theta \right)\mapsto H_{n}\left(\omega ,\theta \right)$ is $F⊗B(\Theta_{n})$-measurable. Observe that, for all ω∈Ω, there exists $\theta \in \Theta_{n}$ such that $H_{n}\left(\omega ,\theta \right)\leq 0$; here we use the fact that $M_{n}$ and *p* are bounded from below. The Sainte-Beuve selection theorem (see Theorem 5.3.2 in [27]) thus implies the existence of a measurable function ${\hat{\theta }}_{n}$ from (Ω,F) to $\left(\Theta_{n},B\left(\Theta_{n}\right)\right)$ such that $H_{n}\left(\omega ,{\hat{\theta }}_{n}\left(\omega \right)\right)\leq 0$ for all ω∈Ω. Clearly, ${\hat{\theta }}_{n}$ must therefore satisfy ${\hat{\theta }}_{n}\left(\omega \right)\in {\hat{\Theta }}_{n}^{*}\left(\omega \right)$ and $p\left({\hat{\theta }}_{n}\left(\omega \right)\right)\leq \inf_{\theta \in {\hat{\Theta }}_{n}^{*}\left(\omega \right)}p\left(\theta \right)+\epsilon_{n}$ for all ω∈Ω, as required. □

**Proof** **of** **Proposition** **8.** We first show that $P^{{\hat{\theta }}_{n}\left(X\right)}\Rightarrow P^{Y}$ as n→∞. For each ω∈Ω, we have ${\hat{\theta }}_{n}\left(\omega \right)\in \Theta_{n}$ and ${\hat{\theta }}_{n}\left(\omega \right)\in {\hat{\Theta }}_{n}^{*}\left(\omega \right)$. These two inclusions justify the following two inequalities respectively:

$$\begin{array}{cc} M({\hat{\theta }}_{n}\left(\omega \right)) & \leq M_{n}\left(\omega ,{\hat{\theta }}_{n}\left(\omega \right)\right)+\underset{\theta \in \Theta_{n}}{\sup}\left|M_{n}\left(\omega ,\theta \right)-M\left(\theta \right)\right|\\ \; & \leq \underset{\theta \in \Theta_{n}}{\inf}M_{n}\left(\omega ,\theta \right)+\lambda_{n}+\underset{\theta \in \Theta_{n}}{\sup}\left|M_{n}\left(\omega ,\theta \right)-M\left(\theta \right)\right|. \end{array}$$

Since $M_{n}\left(\omega ,\theta \right)\leq M\left(\theta \right)+\sup_{\vartheta \in \Theta_{n}}\left|M_{n}\left(\omega ,\vartheta \right)-M\left(\vartheta \right)\right|$ for all $\theta \in \Theta_{n}$, we therefore have

$$M\left({\hat{\theta }}_{n}\left(\omega \right)\right)\leq \underset{\theta \in \Theta_{n}}{\inf}M\left(\theta \right)+\lambda_{n}+2\underset{\theta \in \Theta_{n}}{\sup}\left|M_{n}\left(\omega ,\theta \right)-M\left(\theta \right)\right|.$$

Proposition 2 implies that M(θ)≤M(ϑ)+d(θ,ϑ) for any θ,ϑ∈Θ. Proposition 3 implies that $M(\theta^{†})=0$ for any arbitrary $\theta^{†}\in \Theta^{†}$, so we have $M\left(\theta \right)\leq d\left(\theta ,\theta^{†}\right)$. Consequently, $\inf_{\theta \in \Theta_{n}}M\left(\theta \right)\leq \inf_{\theta \in \Theta_{n}}d\left(\theta ,\theta^{†}\right)$, and so

$$M\left({\hat{\theta }}_{n}\left(\omega \right)\right)\leq \underset{\theta \in \Theta_{n}}{\inf}d\left(\theta ,\theta^{†}\right)+\lambda_{n}+2\underset{\theta \in \Theta_{n}}{\sup}\left|M_{n}\left(\omega ,\theta \right)-M\left(\theta \right)\right|.$$

Integrating both sides over Ω, we obtain

(A3) $$\int_{\Omega }M\left({\hat{\theta }}_{n}\left(\omega \right)\right)dP\left(\omega \right)\leq \underset{\theta \in \Theta_{n}}{\inf}d\left(\theta ,\theta^{†}\right)+\lambda_{n}+2\int_{\Omega }\underset{\theta \in \Theta_{n}}{\sup}\left|M_{n}\left(\omega ,\theta \right)-M\left(\theta \right)\right|dP\left(\omega \right).$$

The first two terms on the right-hand side of (A3) converge to zero as n→∞ under Assumption 5. The third term is $O(\sqrt{V(\Theta_{n})/n})$ by Proposition 6, and therefore must also converge to zero under Assumption 5. Thus we have $\int_{\Omega }M\left({\hat{\theta }}_{n}\left(\omega \right)\right)dP\left(\omega \right)\to 0$ as n→∞. But $\int_{\Omega }M\left({\hat{\theta }}_{n}\left(\omega \right)\right)dP\left(\omega \right)=\int_{\Theta_{n}}M\left(\theta \right)dP^{{\hat{\theta }}_{n}}\left(\theta \right)$, and so Proposition 5 implies that $P^{{\hat{\theta }}_{n}\left(X\right)}\Rightarrow P^{Y}$ as n→∞. We next show that $P\{\omega :p\left({\hat{\theta }}_{n}\left(\omega \right)\right)>\inf_{\theta \in \Theta^{*}}p\left(\theta \right)+\varepsilon \}\to 0$ for any ε>0 as n→∞. Fix ε>0, and choose $\theta^{†}\in \Theta^{†}$ such that $p\left(\theta^{†}\right)\leq \inf_{\theta \in \Theta^{*}}p\left(\theta \right)+\varepsilon /2$. Choose a sequence $\theta_{n}\in \Theta_{n}$, n∈N, such that $d\left(\theta_{n},\theta^{†}\right)=O\left(\inf_{\theta \in \Theta_{n}}d\left(\theta ,\theta^{†}\right)\right)$. Observe that

(A4) $$\begin{array}{ccc} P\left\{\omega :p\left({\hat{\theta }}_{n}\left(\omega \right)\right)>\underset{\theta \in \Theta^{*}}{\inf}p\left(\theta \right)+\varepsilon \right\} & \leq & P\left\{\omega :p\left({\hat{\theta }}_{n}\left(\omega \right)\right)>p\left(\theta^{†}\right)+\varepsilon /2,\;\theta_{n}\in {\hat{\Theta }}_{n}^{*}\left(\omega \right)\right\}\\ & & +P\left\{\omega :\theta_{n}\notin {\hat{\Theta }}_{n}^{*}\left(\omega \right)\right\}. \end{array}$$

We will show that the two terms on the right-hand side of (A4) tend to zero as n→∞. First we consider the first term. If $\theta_{n}\in {\hat{\Theta }}_{n}^{*}\left(\omega \right)$, we must have $p\left({\hat{\theta }}_{n}\left(\omega \right)\right)\leq p\left(\theta_{n}\right)+\epsilon_{n}$. Therefore,

$$\begin{array}{cc} \; & P\left\{\omega :p\left({\hat{\theta }}_{n}\left(\omega \right)\right)>p\left(\theta^{†}\right)+\varepsilon /2,\;\theta_{n}\in {\hat{\Theta }}_{n}^{*}\left(\omega \right)\right\}\\ \; & \leq P\left\{\omega :p\left(\theta_{n}\right)+\epsilon_{n}>p\left(\theta^{†}\right)+\varepsilon /2,\;\theta_{n}\in {\hat{\Theta }}_{n}^{*}\left(\omega \right)\right\}\\ \; & \leq P\left\{\omega :p\left(\theta_{n}\right)+\epsilon_{n}>p\left(\theta^{†}\right)+\varepsilon /2\right\}=1\left\{p\left(\theta_{n}\right)+\epsilon_{n}>p\left(\theta^{†}\right)+\varepsilon /2\right\}. \end{array}$$

Assumption 4 states that *p* is continuous. Therefore, since $d(\theta_{n},\theta^{†})\to 0$ and $\epsilon_{n}\to 0$ as n→∞, we must have $1\{p\left(\theta_{n}\right)+\epsilon_{n}>p\left(\theta^{†}\right)+\varepsilon /2\}=0$ for all sufficiently large *n*. Thus the first term on the right-hand side of (A4) is zero for all sufficiently large *n*. Next we consider the second term on the right-hand side of (A4). We have

$$P\left\{\omega :\theta_{n}\notin {\hat{\Theta }}_{n}^{*}\left(\omega \right)\right\}\leq P\left\{\omega :M_{n}\left(\omega ,\theta_{n}\right)>\lambda_{n}\right\}\leq \lambda_{n}^{-1}\int_{\Omega }M_{n}\left(\omega ,\theta_{n}\right)dP\left(\omega \right),$$

using Markov’s inequality to obtain the second inequality. Proposition 6 gives

$$\begin{array}{cc} \int_{\Omega }M_{n}\left(\omega ,\theta_{n}\right)dP\left(\omega \right) & \leq M\left(\theta_{n}\right)+\int_{\Omega }\underset{\theta \in \Theta_{n}}{\sup}\left|M_{n}\left(\omega ,\theta_{n}\right)-M\left(\theta_{n}\right)\right|dP\left(\omega \right)\\ \; & =M\left(\theta_{n}\right)+O\left(\sqrt{V(\Theta_{n})/n}\right). \end{array}$$

Proposition 2 implies that $M\left(\theta_{n}\right)\leq M\left(\theta^{†}\right)+d\left(\theta_{n},\theta^{†}\right)$, while Proposition 3 implies that $M(\theta^{†})=0$, so we have

$$\begin{array}{cc} \int_{\Omega }M_{n}\left(\omega ,\theta_{n}\right)dP\left(\omega \right) & \leq d\left(\theta_{n},\theta^{†}\right)+O\left(\sqrt{V(\Theta_{n})/n}\right)\\ & =O\left(\underset{\theta \in \Theta_{n}}{\inf}d\left(\theta ,\theta^{†}\right)\right)+O\left(\sqrt{V(\Theta_{n})/n}\right). \end{array}$$

It now follows from Assumption 5 that the second term on the right-hand side of (A4) tends to zero as n→∞. □

**Proof** **of** **Proposition** **9.** We first prove (i). Let $\Lambda_{m}\left(s\right)$ denote the collection of all continuous functions from [0,∞) to R that are linear on each of the m+1 subintervals $\left(0,s_{1}\right),\left(s_{1},s_{2}\right),\ldots ,\left(s_{m},\infty \right)$. $\Lambda_{m}\left(s\right)$ is an (m+2)-dimensional real vector space of functions, and so Theorem 7.2 in [34] implies that $\Lambda_{m}\left(s\right)$ is a VC-major class with $V(\Lambda_{m}\left(s\right))=m+3$. Since $\Psi_{m}\left(s\right)\subset \Lambda_{m}\left(s\right)$, it must be the case that $V\left(\Psi_{m}\left(s\right)\right)\leq V\left(\Lambda_{m}\left(s\right)\right)$. Moreover, given any $\theta_{0}\in \Lambda_{m}\left(s\right)$ and any interval [a,b]⊂R, we can find $\theta_{1}\in \Psi_{m}\left(s\right)$ and c∈R such that $\theta_{1}\left(x\right)=\theta_{0}\left(x\right)+c$ for all x∈[a,b]. It is easy to see that this implies $V\left(\Psi_{m}\left(s\right)\right)\geq V\left(\Lambda_{m}\left(s\right)\right)$. This proves (i). We next prove (ii). First, observe that *d* is a metric (rather than merely a pseudometric) on $\Psi_{m}\left(s\right)$, because any two distinct functions $\theta_{0},\theta_{1}\in \Psi_{m}\left(s\right)$ must differ everywhere on some open interval, which must be of positive $P^{X}$-measure under Assumption 6. Since $F^{Y}$ is strictly increasing on [0,∞) under Assumption 6, we will thus have $F^{Y}\circ \theta_{0}$ and $F^{Y}\circ \theta_{1}$ differing everywhere on the interval in question, forcing $d(\theta_{0},\theta_{1})$ to be nonzero. It remains to show that the metric space $\left(\Psi_{m}\left(s\right),d\right)$ is topologically isomorphic to a complete separable metric space. Each function $\theta \in \Psi_{m}\left(s\right)$ can be written in the form $\theta \left(x\right)=\sum_{i=1}^{m+2}\beta_{i}f_{i}\left(x\right)$ for some unique $\beta \in {\tilde{\Psi }}_{m}\left(s\right)$, where $f_{1}\left(x\right)=1$, $f_{2}\left(x\right)=x$, $f_{i+2}\left(x\right)=\max\left\{0,x-s_{i}\right\}$ for i=1,…,m, and

$${\tilde{\Psi }}_{m}\left(s\right)=\left\{\beta \in R^{m+2}:\sum_{i=1}^{m+2}\beta_{i}f_{i}\left(x\right)\geq 0\;\mathrm{for}\;\mathrm{all}\;x\in R^{X}\right\}.$$

Similarly, each $\beta \in {\tilde{\Psi }}_{m}\left(s\right)$ uniquely identifies a function $\theta \in \Psi_{m}\left(s\right)$. We will denote this bijection between $\Psi_{m}\left(s\right)$ and ${\tilde{\Psi }}_{m}\left(s\right)$ by $S:\Psi_{m}\left(s\right)\to {\tilde{\Psi }}_{m}\left(s\right)$. It is easy to see that ${\tilde{\Psi }}_{m}\left(s\right)$ is a complete separable metric space. We will show that S defines a topological isomorphism between $\left(\Psi_{m}\left(s\right),d\right)$ and $\left({\tilde{\Psi }}_{m}\left(s\right),\tilde{d}\right)$, where $\tilde{d}$ is the usual Euclidean metric on ${\tilde{\Psi }}_{m}\left(s\right)$. That is, we will show that S and $S^{-1}$ are continuous. Suppose $\beta_{1},\beta_{2},\ldots$ is a sequence in ${\tilde{\Psi }}_{m}\left(s\right)$ converging to some $\beta^{*}\in {\tilde{\Psi }}_{m}\left(s\right)$, and let $\theta^{*}=S^{-1}\beta^{*}$ and $\theta_{n}=S^{-1}\beta_{n}$ for each n∈N. For x∈[0,∞), Cauchy’s inequality gives

$$\left|\theta_{n}\left(x\right)-\theta^{*}\left(x\right)\right|=\left|\sum_{i=1}^{m+2}\left(\beta_{n,i}-\beta_{i}^{*}\right)f_{i}\left(x\right)\right|\leq \tilde{d}\left(\beta_{n},\beta^{*}\right){\left(\sum_{i=1}^{m+2}f_{i}{\left(x\right)}^{2}\right)}^{1/2},$$

and hence $\theta_{n}$ converges to $\theta^{*}$ pointwise. It then follows from dominated convergence that $d(\theta_{n},\theta^{*})\to 0$, which proves that $S^{-1}$ is continuous. Suppose now that $\beta_{1},\beta_{2},\ldots$ does not converge to $\beta^{*}\in {\tilde{\Psi }}_{m}\left(s\right)$. Then we can choose a subsequence $\beta_{n_{1}},\beta_{n_{2}},\ldots$ and a constant ε>0 such that $\tilde{d}\left(\beta_{n_{k}},\beta^{*}\right)>\varepsilon$ for all *k*. For $x\in R^{X}$ and all *k*, we have

$$\left|\theta_{n_{k}}\left(x\right)-\theta^{*}\left(x\right)\right|=\tilde{d}\left(\beta_{n_{k}},\beta^{*}\right)\left|\sum_{i=1}^{m+2}\gamma_{n_{k},i}f_{i}\left(x\right)\right|\geq \varepsilon \left|\sum_{i=1}^{m+2}\gamma_{n_{k},i}f_{i}\left(x\right)\right|,$$

where $\gamma_{n_{k}}=\tilde{d}{\left(\beta_{n_{k}},\beta^{*}\right)}^{-1}\left(\beta_{n_{k}}-\beta^{*}\right)$. The subsequence $\gamma_{n_{1}},\gamma_{n_{2}},\ldots$ takes values in the unit sphere in $R^{m+2}$, which is compact, and so we have a further subsequence $\gamma_{n_{k_{1}}},\gamma_{n_{k_{2}}},\ldots$ that converges to some $\gamma^{*}$ in the unit sphere. Therefore, arguing as we did above with Cauchy’s inequality, we have $\sum_{i=1}^{m+2}\gamma_{n_{k_{\ell }},i}f_{i}\left(x\right)\to \sum_{i=1}^{m+2}\gamma_{i}^{*}f_{i}\left(x\right)$ pointwise in *x* as j→∞. Noting that $\sum_{i=1}^{m+2}\gamma_{i}^{*}f_{i}\left(x\right)\neq 0$ on a set of positive $P^{X}$-measure, we conclude that the subsequence $\theta_{n_{k_{\ell }}}$ cannot contain a further subsequence that converges to $\theta^{*}$ pointwise on a set of $P^{X}$-measure one. Recall (see e.g., Theorem 9.2.1 in [35]) that a sequence of random variables converges in probability if and only if every subsequence contains a further subsequence that is almost surely convergent. It must therefore be the case that $\theta_{n}\left(X\right){↛}_{p}\theta^{*}\left(X\right)$. Since $F^{Y}$ has a continuous inverse, this implies that $F^{Y}\left(\theta_{n}\left(X\right)\right){↛}_{p}F^{Y}\left(\theta^{*}\left(X\right)\right)$, which implies that $E|F^{Y}\left(\theta_{n}\left(X\right)\right)-F^{Y}\left(\theta^{*}\left(X\right)\right)|↛0$. That is, $d(\theta_{n},\theta^{*})↛0$. This establishes that S is continuous, which proves (ii). To prove (iii), we must show that (θ,x)↦θ(x) is $B\left(\Psi_{m}\left(s\right)\right)⊗B\left(R^{X}\right)$-measurable. By Theorem 12.2.1 in [35], it suffices to show that $\left(\Psi_{m}\left(s\right),d\right)$ is a separable metric space, and that θ↦θ(x) is continuous in θ for each $x\in R^{X}$. The former assertion was established in (ii). To demonstrate the latter assertion, we let $\theta_{1},\theta_{2},\ldots$ be a sequence in $\Psi_{m}\left(s\right)$ converging to some $\theta^{*}\in \Psi_{m}\left(s\right)$. Using the topological isomorphism S introduced above, we identify our sequence in $\Psi_{m}\left(s\right)$ with another sequence $\beta_{1},\beta_{1},\ldots$ in ${\tilde{\Psi }}_{m}\left(s\right)$ converging to $\beta^{*}\in {\tilde{\Psi }}_{m}\left(s\right)$. As above, we have $|\theta_{n}\left(x\right)-\theta^{*}\left(x\right)|\leq \tilde{d}\left(\beta_{n},\beta^{*}\right){\left(\sum_{i=1}^{m+2}f_{i}{\left(x\right)}^{2}\right)}^{1/2}$ for each $x\in R^{X}$, so that $\theta_{n}$ converges to $\theta^{*}$ pointwise. This proves that θ↦θ(x) is continuous in θ for each $x\in R^{X}$. □

**Proof** **of** **Proposition** **10.** We first show that $\Theta^{†}$ is dense in $\Theta^{*}$ under *d*. Fix a function $\theta^{*}\in \Theta^{*}$. The function ${\tilde{\theta }}^{*}=F^{Y}\circ \theta^{*}\circ Q^{X}$ is a Borel measurable mapping from [0,1) to [0,1); we extend the domain and range of ${\tilde{\theta }}^{*}$ to [0,1] by setting ${\tilde{\theta }}^{*}\left(1\right)=1$. A well known consequence of Urysohn’s lemma (see e.g., Lemma 2.6.3 in [35]) is that the continuous functions on [0,1] form a dense subset of the Lebesgue integrable functions on [0,1] under the $L_{1}$-seminorm. It is also well known (see e.g., Theorem 11.2.4 and the following example in [35]) that any continuous function on [0,1] can be approximated arbitrarily well by a continuous piecewise linear function on [0,1], with finitely many kinks. Such a function can in turn be approximated arbitrarily well by a continuous piecewise linear function on [0,1], with finitely many kinks, for which the slope of the function is nonzero wherever it is defined. We will let ${\tilde{\theta }}_{1},{\tilde{\theta }}_{2},\ldots$ be a sequence of such functions, chosen such that $\lim_{k\to \infty }\int \left|{\tilde{\theta }}^{*}\left(u\right)-{\tilde{\theta }}_{k}\left(u\right)\right|du=0$ and $0\leq {\tilde{\theta }}_{k}\leq 1$ for each k∈N. Let *U* be a random variable distributed uniformly on [0,1], and let $F^{U}\left(\cdot ;{\tilde{\theta }}_{k}\right)$ denote the distribution function of ${\tilde{\theta }}_{k}\left(U\right)$. The piecewise linearity and nonzero slope of each ${\tilde{\theta }}_{k}$ ensures that $F^{U}\left(\cdot ;{\tilde{\theta }}_{k}\right)$ is Lipschitz continuous for each *k*. Specifically, for a,b∈[0,1] we have $|F^{U}\left(b;{\tilde{\theta }}_{k}\right)-F^{U}\left(a;{\tilde{\theta }}_{k}\right)|\leq \nu_{k}^{-1}\left(N_{k}+1\right)\left|b-a\right|$, where $\nu_{k}$ is a lower bound on the derivative of ${\tilde{\theta }}_{k}$, and $N_{k}$ is the number of kinks. Let ${\tilde{\theta }}_{k}^{†}:\left[0,1\right]\to \left[0,1\right]$ be defined by ${\tilde{\theta }}_{k}^{†}\left(u\right)=F^{U}\left({\tilde{\theta }}_{k}\left(u\right);{\tilde{\theta }}_{k}\right)$. Since ${\tilde{\theta }}_{k}$ and $F^{U}\left(\cdot ;{\tilde{\theta }}_{k}\right)$ are Lipschitz continuous for each *k*, each ${\tilde{\theta }}_{k}^{†}$ must also be Lipschitz continuous. Let $\theta_{k}^{†}=Q^{Y}\circ {\tilde{\theta }}_{k}^{†}\circ F^{X}$, and observe that $\theta_{k}^{†}\left(X\right)=Q^{Y}\left({\tilde{\theta }}_{k}^{†}\left(F^{X}\left(X\right)\right)\right)∼Q^{Y}\left({\tilde{\theta }}_{k}^{†}\left(U\right)\right)=Q^{Y}\left(F^{U}\left({\tilde{\theta }}_{k}\left(U\right);{\tilde{\theta }}_{k}\right)\right)∼Q^{Y}\left(U\right)∼Y$. This establishes that $\theta_{k}^{†}\in \Theta^{†}$ for each *k*. It remains to show that $\lim_{k\to \infty }d\left(\theta_{k}^{†},\theta^{*}\right)=0$. Since $d\left(\theta_{k}^{†},\theta^{*}\right)=\int \left|{\tilde{\theta }}_{k}^{†}\left(u\right)-{\tilde{\theta }}^{*}\left(u\right)\right|du$, it suffices to show $\lim_{k\to \infty }\int \left|{\tilde{\theta }}_{k}^{†}\left(u\right)-{\tilde{\theta }}_{k}\left(u\right)\right|du=0$. Observe that $\int |{\tilde{\theta }}_{k}^{†}\left(u\right)-{\tilde{\theta }}_{k}\left(u\right)|du=\int |F^{U}\left({\tilde{\theta }}_{k}\left(u\right);{\tilde{\theta }}_{k}\right)-{\tilde{\theta }}_{k}\left(u\right)|du\leq \sup_{0\leq v\leq 1}\left|F^{U}\left(v;{\tilde{\theta }}_{k}\right)-v\right|$. Since ${\tilde{\theta }}^{*}\left(U\right)∼U$ and $\lim_{k\to \infty }\int \left|{\tilde{\theta }}^{*}\left(u\right)-{\tilde{\theta }}_{k}\left(u\right)\right|du=0$, Propositions 2–4 jointly imply that ${\tilde{\theta }}_{k}\left(U\right)\to_{d}U$ as k→∞. But this implies that $\lim_{k\to \infty }\sup_{0\leq v\leq 1}\left|F^{U}\left(v;{\tilde{\theta }}_{k}\right)-v\right|=0$, which proves $\lim_{k\to \infty }\int \left|{\tilde{\theta }}_{k}^{†}\left(u\right)-{\tilde{\theta }}_{k}\left(u\right)\right|du=0$. We conclude that $\Theta^{†}$ is dense in $\Theta^{*}$ under *d*. Next we show that $\inf_{\theta \in \Theta_{n}}d\left(\theta ,\theta^{†}\right)=O\left(m_{n}\sup_{0\leq i\leq m_{n}}P^{X}{\left(s_{i,n},s_{i+1,n}\right)}^{2}\right)$ for each $\theta^{†}\in \Theta^{†}$. Choose $\theta_{n}\in \Theta_{n}$ such that $\theta_{n}\left(s_{i,n}\right)=\theta^{†}\left(s_{i,n}\right)$ for $i=0,\ldots ,m_{n}$, and such that $\theta_{n}\left(x\right)$ is constant for $x\geq s_{m_{n},n}$. Then we have

$$\begin{array}{ccc} d(\theta_{n},\theta^{†}) & = & \sum_{i=0}^{m_{n}}\int_{s_{i,n}}^{s_{i+1,n}}\left|F^{Y}\left(\theta_{n}\left(x\right)\right)-F^{Y}\left(\theta^{†}\left(x\right)\right)\right|dF^{X}\left(x\right)\\ & = & \sum_{i=0}^{m_{n}}\int_{F^{X}\left(s_{i,n}\right)}^{F^{X}\left(s_{i+1,n}\right)}\left|F^{Y}\circ \theta_{n}\circ Q^{X}\left(u\right)-F^{Y}\circ \theta^{†}\circ Q^{X}\left(u\right)\right|du, \end{array}$$

where $F^{X}\left(\infty \right)=1$. Our choice of $\theta_{n}$ and the Lipschitz property of $F^{Y}\circ \theta^{†}\circ Q^{X}$ ensure that

$$\underset{F^{X}\left(s_{i,n}\right)<u<F^{X}\left(s_{i+1,n}\right)}{\sup}|F^{Y}\circ \theta_{n}\circ Q^{X}\left(u\right)-F^{Y}\circ \theta^{†}\circ Q^{X}\left(u\right)|\leq K|F^{X}\left(s_{i+1,n}\right)-F^{X}\left(s_{i,n}\right)|$$

for $i=0,\ldots ,m_{n}$, where *K* is the Lipschitz coefficient of $F^{Y}\circ \theta^{†}\circ Q^{X}$. We thus have

$$\begin{array}{cc} d(\theta_{n},\theta^{†}) & \leq K\sum_{i=0}^{m_{n}}{\left|F^{X}\left(s_{i+1,n}\right)-F^{X}\left(s_{i,n}\right)\right|}^{2}\\ \; & =K\sum_{i=0}^{m_{n}}P^{X}{\left(s_{i,n},s_{i+1,n}\right)}^{2}\leq Km_{n}\underset{0\leq i\leq m_{n}}{\sup}P^{X}{\left(s_{i,n},s_{i+1,n}\right)}^{2}, \end{array}$$

giving the desired result. □
